# Deep pemphigus (pemphigus vulgaris, pemphigus vegetans and paraneoplastic pemphigus) in dogs, cats and horses: a comprehensive review

**DOI:** 10.1186/s12917-020-02677-w

**Published:** 2020-11-23

**Authors:** Heng L. Tham, Keith E. Linder, Thierry Olivry

**Affiliations:** 1 Department of Small Animal Clinical Sciences, Virginia-Maryland College of Veterinary Medicine, Virginia Tech, Blacksburg, VA USA; 2 Comparative Medicine Institute, College of Veterinary Medicine, North Carolina State University, Raleigh, NC USA; 3 Department of Population Health and Pathobiology, College of Veterinary Medicine, North Carolina State University, Raleigh, NC USA; 4 Department of Clinical Sciences, College of Veterinary Medicine, North Carolina State University, Raleigh, NC USA

**Keywords:** Pemphigus, Vulgaris, Vegetans, Paraneoplastic, Desmoglein, Suprabasal, Acantholysis, Canine, Feline, Equine

## Abstract

Pemphigus is the term used to describe a group of rare mucocutaneous autoimmune bullous diseases characterized by flaccid blisters and erosions of the mucous membranes and/or skin. When the autoantibodies target desmosomes in the deep layers of the epidermis, deep pemphigus variants such as pemphigus vulgaris, pemphigus vegetans and paraneoplastic pemphigus develop. In this article, we will review the signalment, clinical signs, histopathology and treatment outcome of pemphigus vulgaris, pemphigus vegetans and paraneoplastic pemphigus in dogs, cats and horses; where pertinent, we compare the animal diseases to their human homologue. Canine, feline and equine pemphigus vulgaris, pemphigus vegetans and paraneoplastic pemphigus have many features similar to the human counterpart. These chronic and often relapsing autoimmune dermatoses require aggressive immunosuppressive therapy. In animals, the partial-to-complete remission of pemphigus vulgaris and pemphigus vegetans has been achieved with high dose glucocorticoid therapy, with or without adjunct immunosuppressants; the prognosis is grave for paraneoplastic pemphigus.

## Background

Desmosomes are intercellular adhesion structures that are well developed in tissues that experience considerable mechanical stress, such as the epidermis, mucosae, and myocardium. Desmosomes maintain cell-to-cell structural integrity, anchor internal cytoskeletal intermediate filaments, and participate in many functions, such as signaling, differentiation, inflammation and carcinogenesis [1]. Diseases that disrupt or weaken desmosomes may lead to loss of cell-cell adhesion, a process called acantholysis [1]. In animals, the spectrum of skin diseases that can lead to acantholysis is wide, ranging from genetic acantholytic dermatoses (i.e., suprabasal epidermolysis bullosa simplex in cattle) to infectious proteolytic acantholytic dermatoses (i.e., exfoliative superficial pyodermas in dogs), and finally to autoimmune acantholytic dermatoses (i.e., pemphigus variants) [2].

Pemphigus is the term used to describe a group of rare mucocutaneous autoimmune bullous diseases characterized by flaccid blisters and/or pustules, with secondary erosions of the mucous membranes and/or skin [3, 4]. In these diseases, autoantibodies (AA) most often target specific epidermal and/or mucosal desmosomal adhesion proteins required to maintain the strength of keratinocyte intercellular adhesion [5], which leads to a loss of cellular adhesion, blister formation and clinical lesions. The expression pattern of desmoglein (DSG)-1 and DSG3 is different between, and within, the skin and mucosae. In the skin, DSG1 is expressed throughout the epidermis, but more intensely in the superficial layers, while DSG3 is expressed in the deeper layers (i.e., basal layer). In contrast, while DSG1 and DSG3 are expressed throughout the mucosae, the latter is expressed at much higher intensity than the former [6]. The type and severity of clinical disease that develops depends on the type of antidesmosomal AA (i.e., anti-DSG3 ± anti-DSG1), as well as the location and relative amount of desmosomes targeted by AA. For example, when AA target proteins within superficially expressed desmosomes (i.e., DSG1 and/or desmocollin [DSC]-1) restricted to the granular and/or upper spinous layers of the epidermis, a form of superficial pemphigus known as pemphigus foliaceus (PF) develops [5] that only affects the skin, and not the mucosae. The superficial pemphigus diseases of animals were reviewed previously [7, 8]. In contrast, when the AA target desmosomes in the deep layers of the mucosae (i.e., DSG3), deep erosive and vesicular, mucosal-predominant variant of pemphigus (i.e., pemphigus vulgaris [PV]) arises [9]. The mucocutaneous form of PV is associated with autoantibodies against both DSG3 and DSG1 [5].

In this literature review, we focus on the three types of deep pemphigus recognized in animals: PV, pemphigus vegetans (PVeg) and paraneoplastic pemphigus (PNP). We review the available information published to date and, where relevant, compare the animal disease to the human homologue. To ensure that the diagnoses for these three forms of deep pemphigus in animals are correct and, as accurately as possible, reflect the human counterpart, we defined the inclusion criteria for published pemphigus cases based on the consensus guidelines in human medicine [3, 4].

### Search strategy and case selection

A literature search for any case reports or series of PV, PVeg and PNP in dogs, cats and horses was conducted on the 21st and 22nd of October 2019 using four online databases: PubMed, Web of Science Core Collection (Clarivate Analytics), CAB Abstract (from CAB Direct) and Google Scholar (scholar.google.com). There was no language or date restriction placed on the search. The bibliography of selected reports was reviewed for any additional publications not captured by search engine strategies. The following search strategy was used for these three databases:

((dog OR dogs OR canine) OR (cat OR cats OR feline) OR (horse* OR equine)) AND ((autoimmune OR immune-mediated AND skin) OR ((pemphigus OR penfigo) AND (vulg* OR paraneoplastic OR veg*)))

The search strategies used for Google Scholar, which does not use a Boolean logic, were as follows:

- Pemphigus vulgaris: *pemphigus vulgaris [species 1]*^*¶*^*, [species 2]*^*ǂ*^ *pemphigus vulgaris*
- Pemphigus vegetans: *pemphigus vegetans [species 1]* ^*¶*^*, [species 2]* ^*ǂ*^ *pemphigus vegetans*
- Paraneoplastic pemphigus: *paraneoplastic pemphigus [species 1]* ^*¶*^*, [species 2]* ^*ǂ*^ *paraneoplastic pemphigus*

^¶^ dog or cat or horse

^ǂ^ canine or feline or equine

Excluded were review publications without specific clinical case information, proceedings, and abstracts due to the lack of detailed original case material needed to meet the inclusion criteria and to ensure quality material for review.

Case reports or series of canine, feline and equine PV, PVeg and PNP were included in this review if they fulfilled the following inclusion criteria [3, 4]:

- *Pemphigus vulgaris*

1. A clinical presentation that included mucosal and/or cutaneous vesicles/bullae and/or deep erosions and/or ulcers, AND
2. A histopathology demonstrating, at least in some sections, suprabasal epidermal, mucosal, or follicular acantholysis

- *Pemphigus vegetans*

1. A clinical presentation manifested by mucocutaneous or cutaneous verruciform (that is “wart-like”) or vegetative plaques, and/or pustular skin lesions, with or without mucosal and/or cutaneous vesicle/bullae, and/or deep erosions and/or ulcers, AND
2. A histopathology demonstrating features of PV-type suprabasal acantholysis and epidermal hyperplasia, with or without intraepidermal neutrophilic and/or eosinophilic acantholytic pustules

- *Paraneoplastic pemphigus*

1. A clinical presentation of mucosal or mucocutaneous erosions and/or ulcers, AND
2. A histopathology of suprabasal acantholysis and lymphocyte-mediated interface dermatitis and apoptotic keratinocytes at all epidermal levels, AND
3. The demonstration of a concomitant malignant neoplasia at the time, or after the development of mucosal and/or cutaneous lesions

A positive direct (DIF) or indirect immunofluorescence (IIF) test was not an inclusion criterion because, unlike humans, a study on canine PF showed that a positive DIF or IIF is not specific for pemphigus [10]. Additionally, and unlike their human counterpart, DIF or IIF testing for animal pemphigus is not readily available in veterinary medicine, and, whenever available, it has been used only for research purposes and not as an ancillary diagnostic tool.

### Case selection outcome

**Fig. 1:**
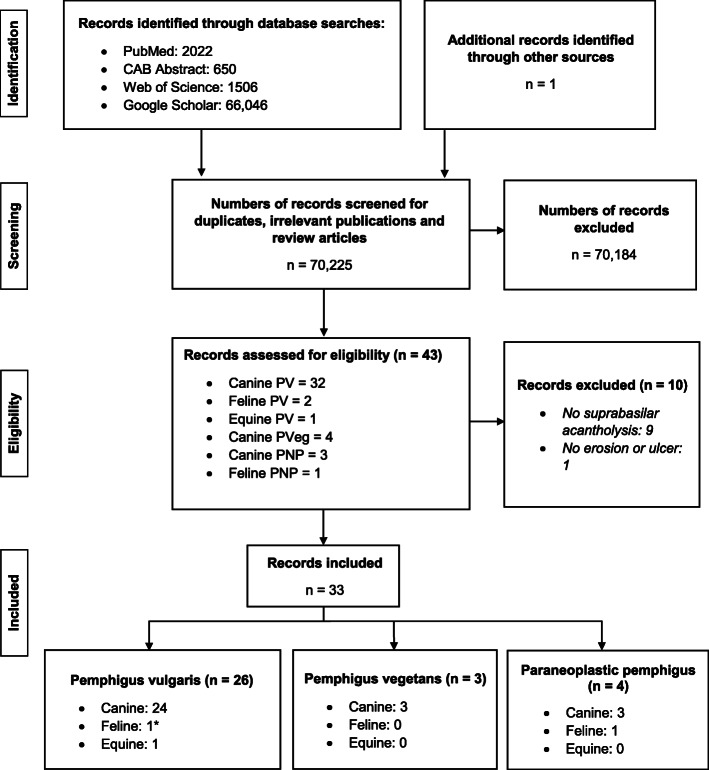
Literature search strategy. Flow diagram of study selection. *Several papers reported the same cats with PV and data that are not duplicated are pooled for analysis in this review. The numbers correspond to published papers, not animals

The search of PubMed, CAB Abstract, Web of Science and Google Scholar yielded a total of 70,225 records (Fig. 1). One record [11] was identified via the author’s publication list from a review paper [12]. Among all of the records identified, 43 were assessed for eligibility, of which 10 [13–22] records were excluded because of lack of fulfilment of inclusion criteria (Fig. 1). The remaining 33 records are reviewed herein.

### Pemphigus vulgaris

#### Introduction

Pemphigus vulgaris is a chronic autoimmune blistering dermatosis that affects the mucosal, mucocutaneous junction and/or skin; the mucosal and/or skin lesions evolve from flaccid blisters (vesicles and/or bullae) to deep erosions, and are associated with pain, especially when the lesions develop in the oral cavity [3]. Based on the latest recommendations by an international panel of experts on human pemphigus [4], the diagnosis of PV requires the aforementioned clinical presentation and a histopathology that demonstrates an intraepithelial suprabasal acantholysis, and either a positive DIF or the serological detection of AA against epithelial cell surface antigens.

#### Historical perspective

The word ‘pemphigus’ was first used by Hippocrates (460–370 BC) to describe a terrible-in-appearance disease with fever, which he, back then, referred to as *pemphigoides pyretoi* (cited in [23]). However, Hippocrates did not give any valuable description of the disease and therefore, the exact etiopathogenesis of what he observed is not known. The first author to describe a disease in a patient consistent with human PV was MacBride in 1777 (cited in [24]). Lever was the first physician to describe the clinical and histopathological features of PV in detail, which then was referred to as *pemphigus vulgaris malignus* [24]. Readers interested in the history of human pemphigus and pemphigoid in humans are referred to two articles published by Lever [23, 24], which were later reviewed in 1979 [25].

The first case series of canine PV can be traced back to two papers published in 1975 in the same journal issue – these were authored by Hurvitz and Feldman [26] and Stannard et al [27]. Hurvitz reported five dogs with a disease resembling human PV; however, based on the inclusion criteria followed herein for animal PV, only one dog (case 1) could be confirmed as having PV, because suprabasal epidermal acantholysis was not documented in the remaining four dogs. In Stannard’s report of three dogs, only two dogs fit our inclusion criteria for PV – the one remaining dog (case 1) most likely had PNP; this dog will be discussed in the PNP section below. Following these two case series of canine PV, numerous case reports were published over the following three decades, with the most recent report published in 2018 [28].

Reports on feline PV are even rarer than those of the canine disease. The first published report of putative feline PV was in 1979 by Brown and Hurvitz [20]. However, it is unlikely that the cat’s skin and oral lesions were due PV because of incompatible clinical lesions and the absence of documentation of suprabasal epidermal or follicular acantholysis – this case is not included herein. In 1980, Scott reported PV in a castrated male domestic shorthaired (DSH) cat [29] and, in our opinion, this is likely the first published case of feline PV that fits our inclusion criteria.

Equine PV was first anecdotally reported in 2000 [30], and this was followed by several mentions in textbooks [31]. To date, there is only one detailed case report of equine PV in a Welsh pony stallion [31].

Other than in the dog, cat and horse, PV has also been reported in a pigtail macaque [32] and a llama [33]. However, these case reports are not included in this review.

#### Incidence and prevalence

Pemphigus vulgaris is the most common clinical pemphigus variant in human [9], and it corresponds to 70% of cases of pemphigus seen in France [34]. Another paper specifies that PV consists of almost 90% of 180 patients with pemphigus seen at a tertiary referral dermatology center in Israel between 2000 and 2015 [35]. In contrast, a recent paper highlights that pemphigus foliaceus/erythematosus is more common than PV in northern Finland [36]. The reason for this disparity is unknown, but this could be due to a small study population or to regional genetic differences. The incidence varies from 0.8 to 16.1 new cases per million per year, depending on the geographical area and ethnicity [9]. The exact global prevalence of human PV is unknown, but some regional information does exist: for example, the prevalence is reported as 94.8 patients/million population in Germany [37].

There are no available data to estimate the global or regional incidence and prevalence of canine, feline or equine PV. However, out of 9750 dogs and 2050 cats examined for skin diseases at the New York State College of Veterinary Medicine between 1975 and 1984, PV accounted for 0.1 and 0.2% of canine and feline dermatoses examined at this veterinary teaching hospital, respectively [38]. The authors also stated that canine and feline PV was the second most common form of pemphigus seen at their institution. Most recently, in a retrospective study of 85 canine skin samples diagnosed with an autoimmune disease between 2004 and 2016, PV was found in two dogs (2%) [39]. These data, albeit scarce, indicate that unlike their human counterpart, PV is one of the rarest autoimmune dermatosis in animals.

#### Etiopathogenesis

In human PV, DSG3 and DSG1 are the main autoantigens targeted by immunoglobulin G (IgG) AA [9]. Patients affected by mucosal-dominant PV only have detectable anti-DSG3 IgG AA [9], whereas those with mucocutaneous form of PV have both anti-DSG3 and DSG1 IgG AA [40]. There are several theories proposed to explain the pathogenesis of blister formation in PV, which may coexist: the DSG1/DSG3 compensation, steric hindrance, desmoglein internalization/disassembly theories [41] and Grando’s apoptolysis hypothesis [42]. Other papers have suggested that IgG-independent factors (i.e., T helper (Th) 2 cytokines, tumor necrosis factor-alpha (TNF-α) and Fas ligand) do play a role in the pathogenesis of acantholysis in PV [9]. Readers are referred to articles published elsewhere for more detailed information of the aforementioned theories of blister formation in pemphigus [5, 41–44].

**Fig. 2:**
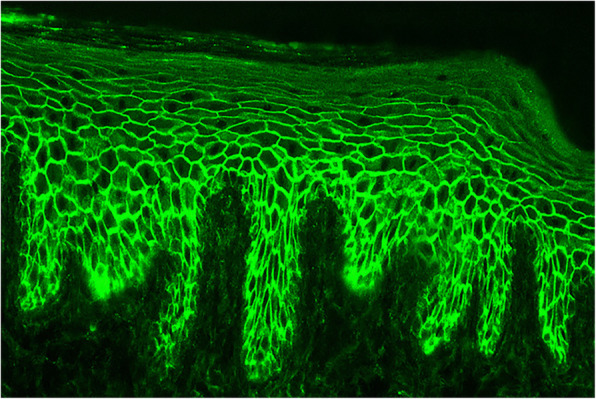
Autoantibody detection in canine pemphigus vulgaris. Indirect immunofluorescence performed on a canine buccal mucosal substrate revealed a “fishnet pattern” suggesting the presence of anti-keratinocyte IgG auto-antibodies in the serum of a dog with pemphigus vulgaris (courtesy of Thierry Olivry)

In animals, the first study to attempt to localize the canine PV antigen(s) was published by Suter in 1990 [45]. In this study, immunohistochemistry and immunoelectron microscopy utilized canine esophagus and cultured canine keratinocyte substrates and sera from human patients with pemphigus (one from a patient with PV; the remaining from humans with PF). Results showed that human pemphigus AA reacted with the canine interdesmosomal cytoplasmic membrane components. Although the findings of this study indicated that both canine substrates expressed antigen(s) recognized by human PV AA, it was not evidence that canine PV was due to circulating AA. It was not until 2003 that Olivry and colleagues, by means of immunofluorescence, immunoblotting and immunoprecipitation, detected the presence of anti-keratinocyte AA in the serum (Fig. 2) and lesional skin of dogs with PV and documented for the first time that DSG3 was also the major canine PV antigen [46]. This evidence was soon supported by Nishifuji and colleagues who confirmed that human and canine PV serum AA bound to the extracellular domains of canine DSG3 [47]. A third paper verified these findings in 2007, reporting that the AA were pathogenic as they were able to dissociate keratinocytes in culture [6]. Finally, neonatal mice that were injected intradermally with serum IgG from dogs with PV developed suprabasal acantholytic blisters, further supporting the role of circulating IgG AA in the pathogenesis of canine PV [10].

In addition to anti-DSG3 AA, Williamson and colleagues reported the upregulation of c-Myc in the perilesional and nonlesional epidermis and oral mucosa of two dogs diagnosed with PV and one with coexisting PV/PF [48]. The proto-oncogen c-Myc is known to regulate the proliferation and terminal differentiation in mammalian cells [49]. The authors suggested that PV antibodies, and consequently high levels of c-Myc, interfere with the signaling cascade controlling the expression of DSG3 in basal and immediate suprabasal keratinocytes, which may contribute to epidermal stem cell depletion and the persistence of lesion in human patients with PV. Finally, in one report [50], the application of polymyxin B ear drops was suspected to induce the onset of PV in a dog, but the drug-induced nature of this case was challenged soon thereafter [51].

There is very little information on the immunopathogenesis of feline PV. Four cats with PV reported by Scott et al. [38] were found to have intercellular epidermal deposition of immunoglobulins via direct immunofluorescence, with one cat also having deposition of activated complement fraction 3 (C3); none of these cats had detectable circulating AA.

We reported the presence of circulating anti-keratinocyte IgG AA using equine lip as substrate in the only single report of equine PV [31]. Immunoblotting and immunoprecipitation confirmed that this horse’s AA recognized the extracellular domains of both canine DSG3 and DSG1 [31]. Whether these AA would recognize also equine DSG3 and DSG1 is logical to expect, but it is not proven.

#### Signalment

In humans, a high incidence of PV is observed in Ashkenazi Jews [52], with one study reporting that PV is 3.6 times more frequent among Jews as compared to Arabs in Israel [53]. The mean age of onset is between 40 to 60 years and the age at presentation of human patients with PV ranges from 36 to 72 years [9, 52]. The female-to-male ratio in Americans with PV is estimated to be 5.0 [9], which implies that PV is five times more common in American females than males. Other reports estimated a lower female-to-male ratio of 1.5 to 4.0 but the consensus is that PV is more prevalent among women [54].

The following breed information is derived from 24 publications including 54 dogs with PV [11, 26–28, 38, 46, 50, 55–71]. We noted that the German shepherd mixed breed dog and the Weimaraner reported by Scott and colleagues in 1982 [70] are the same dogs –case 10 and 11, respectively—reported by the authors in 1987 (Danny Scott, personal communication) [38]. The German shorthaired pointer in Bensignor’s 1998 case report [61] was also included in the case series published in 2000 by Carlotti and colleagues [60]. Additionally, three dogs with PV in the immunology paper by Olivry and colleagues in 2002 [46] came from other reports [58, 59]. Altogether, there were 10 crossbred dogs (19%), nine German shepherd/German shepherd crossbred dogs (17%), six Collie-associated breeds (five Collies and one Border collie) (11%), four Spaniels (two Cocker spaniels, one springer spaniel and one unspecified) (7%), two Dachshunds (4%), Labrador retrievers (4%), Poodles (4%) and Scottish terriers (4%), and one each (2%) of the following: Australian shepherd dog, Belgian shepherd dog, Chesapeake Bay retriever, Dalmatian, Doberman, German shorthaired pointer, great Dane, Howavart, Irish setter, miniature Schnauzer, Shetland sheepdog, Spanish mastiff, Tosa Inu and a Weimaraner. There were three dogs in which the breed was not specified [11, 63, 66]. Gender information was available for 51 PV-affected dogs [26–28, 38, 46, 50, 55–62, 64, 65, 67–71] yielding a female-to-male ratio of 0.6; male dogs seem more often affected than females, which is the opposite of human PV. The mean and median ages of onset were 6 and 7 years, respectively (range: 8 months to 14 years). More than 70% (36/51) of dogs had a disease onset at, or after, 5 years, which is thought to correspond to a similar middle-age of onset in humans [26–28, 38, 46, 55, 56, 58–62, 64, 67–71].

To date, signalment information on feline PV is only available for four cats [38]. It is likely that the cat in Scott’s 1980 paper [29] was also included in subsequent papers published by the same author in 1980 [72], 1984 [73] and 1987 [38], and by Manning et al in 1982 [74]. All four cats with PV were domestic shorthairs; one was female and three were males. The age when PV was diagnosed ranged from 1 to 14 years old (median: 5 years).

The only horse with PV reported in the literature was a 9-year-old Welsh pony stallion [31]. The onset of skin lesions occurred 3 months prior to presentation to the veterinarian.

#### Clinical signs

In humans, PV most commonly presents with a mucosal or a mucocutaneous phenotype [54]. The course of the disease often begins with oral lesions [3, 54], which are reported to be the first manifestation in 50 to 70% of cases, and affect 90% of patients during the course of their disease [54]. In some individuals, oral lesions might be the only clinical manifestation [54]. If there is cutaneous involvement, the skin lesions usually appear several weeks or months after the onset of mucosal lesions [3]. The most commonly affected oral regions are the buccal and palatine mucosae, lips and gingivae, and the lesions may extend to the vermilion border of the lips [54]. These oral lesions are often painful and interfere with eating [3]. Cutaneous lesions tend to predominate at “seborrheic” areas, such as the chest, face, scalp, and interscapular region [3]. The oral lesions are usually characterized by secondary deep erosions and rarely the primary vesicle or bullae are observed, because the blisters are fragile and rupture easily [54]. Cutaneous lesions present as flaccid vesicles and bullae with a clear serous fluid content that often rupture quickly to form deep erosions [3]; these lesions may develop on normal or erythematous skin [9, 54]. As these erosive lesions evolve, they become covered by crusts and tend not to heal. If there is scalp involvement, scaly plaques and alopecia can also develop [54]. Nails are rarely affected in human PV, but this phenomenon has been documented in several patients [75–77] with one report stating that 20% of patients have only the nails affected [78]. Pemphigus vulgaris is usually not associated with a strong pruritus [3], but it has been reported in humans [79, 80]. A marginal Nikolskiy sign (i.e., an epidermal detachment caused by mechanical pressure at the edge of a vesicle or normal skin) is usually present in PV [34, 54]. Human PV is a chronic disease, and secondary bacterial infections are the most common complications that can lead to septic shock [54]. Additionally, oral lesions are very painful, impair food intake, and have a negative impact on the nutritional status of the patient [9, 54].

For this review, we considered the canine and feline nasal planum as a modified mucosa, and the ears (concave/medial/inner and convex/lateral/outer pinnae) and haired muzzle as non-mucosal (i.e., cutaneous) skin. Information on the primary complaint(s) at patient presentation to the veterinarian was available for 21 dogs [11, 26–28, 50, 55–59, 61–65, 68, 70, 71], of which seven (33%) presented for mucocutaneous lesions [50, 57, 58, 62, 64, 65], four (19%) for oral lesions (including lips) only [27, 61, 68], four (19%) for non-dermatological signs, such as hypersalivation, halitosis, and/or dysphagia [11, 26, 59, 71], and three (14%) for skin-restricted lesions (including paw pads) [55, 56, 70]. The remaining three cases presented for onychomadesis [70], a pruritic dermatitis [63] and a combination of oral lesions, inappetence and lethargy [28].

**Fig. 3:**
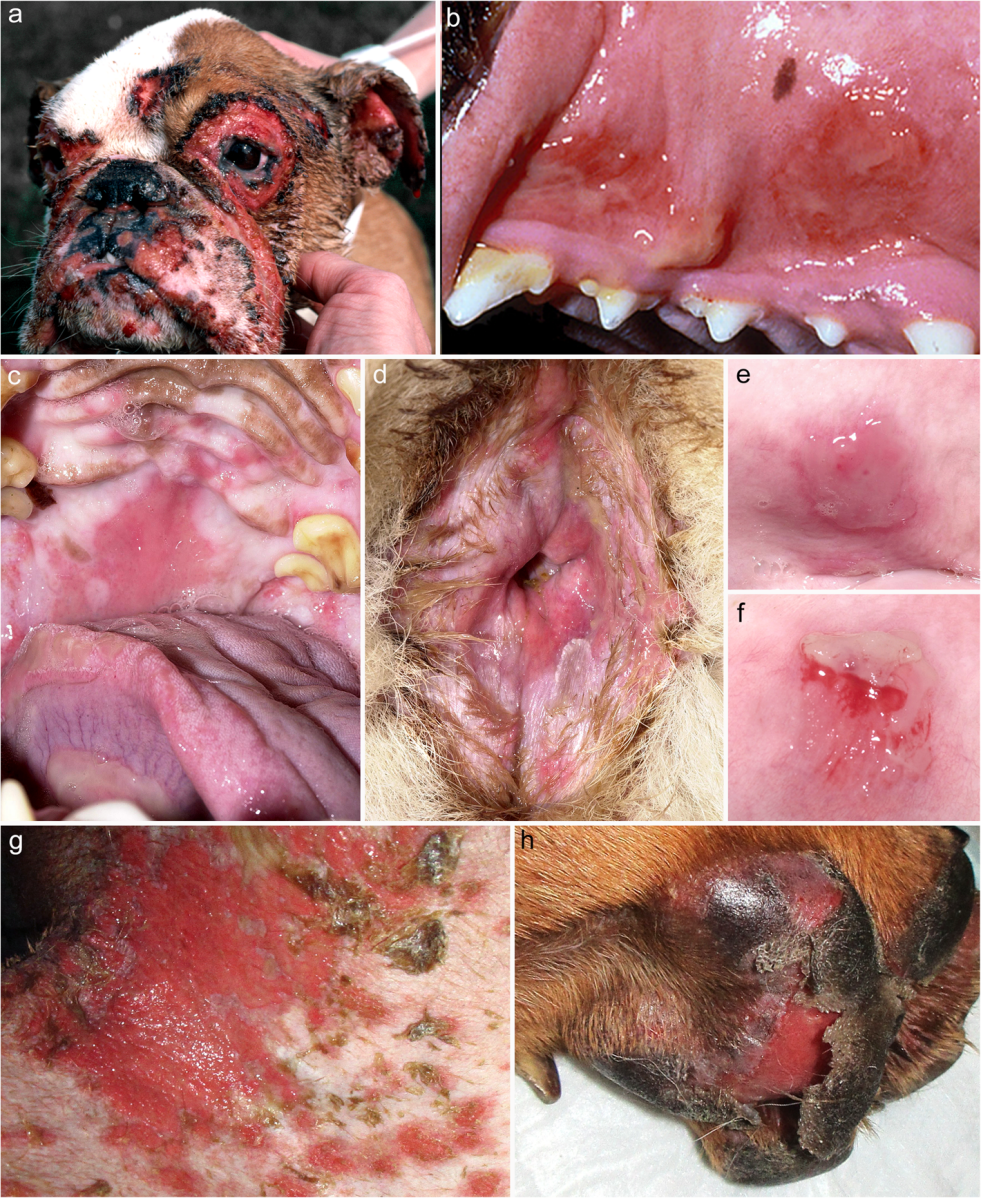
Clinical characteristics of canine pemphigus vulgaris. **a** bilaterally symmetrical erosions and ulcerations on the face of an English bulldog (courtesy of Margreet Vroom); **b** desquamative and erosive stomatitis – the mucosal epithelium is seen detaching from underlying erosions on the gingiva (courtesy of Stephen D. White); **c** erosive and ulcerative stomatitis affecting the palate; d: erosive and exfoliative anitis; **e** flaccid vesicle on the gingiva; **f** same lesion as in (**e**) after exfoliation of the epithelium – c-to-f were taken from the same German shepherd dog (courtesy of Thierry Olivry); **g**: widespread erosive dermatitis on the groin; **h**: sloughing of the footpad – **g** and h were from the same dog (courtesy of Nina Thom)

Out of 54 cases of canine PV, 50 dogs (93%) exhibited a mucosal or mucocutaneous phenotype, of which the lips and/or oral cavity were the most commonly affected regions (46/50 dogs, 92%). Three dogs (6%) only had oral cavity (including lips) involvement [27, 60, 67], two (4%) had only skin lesions [60, 70] and two (4%) had only the nails/claws affected [60, 70]. The involvement of claws (nails), alone or with other regions, was reported in four dogs (7%), all four exhibiting onychomadesis [26, 60, 61, 70]. Unusually, one dog that initially only had skin lesions, developed oral lesions when the disease relapsed for the second time [64]. In 41/44 dogs (93%) in which the distribution was specified, there was involvement of the head or face (Fig. 3) (i.e., the oral cavity and/or lips, nasal planum/nose, perinasal/muzzle, periocular and/or pinnae). In 32 dogs (59%) with oral cavity lesions [11, 26–28, 46, 50, 55, 56, 58–63, 65, 66, 68, 69, 71], the gingiva and palate were the two most commonly affected regions followed by the tongue. One dog had almost the entire oral cavity involved [26]. In the two dogs with skin-restricted lesions, the affected regions were the dorsal muzzle [70] and paws, including the pads [60]. The cutaneous lesions, including those of the paws, pawpads and nails, were bilateral and symmetrical in 15 dogs [27, 28, 50, 55–58, 61, 62, 64, 68–71] with the symmetry not specified in the others.

The most common lesion types in canine PV are deep erosions and shallow ulcers followed by (in a descending order) crusts, vesicles, erythema, depigmentation and alopecia. These lesions are very similar to those of human PV. As in humans, canine PV is predominantly a non-pruritic dermatosis, with pruritus reported only in one dog [63] in which it was most likely due to a concurrent superficial pyoderma. A positive marginal Nikolskiy sign was only reported in three dogs [50, 56, 63]. Systemic signs were reported in 19/54 dogs (35%) [11, 26–28, 38, 50, 55, 56, 61, 62, 65, 68, 70, 71], and they consisted of lymphadenopathy, lethargy/dullness, anorexia, pain, weight loss, diarrhea and/or hyperthermia. In dogs in whom pain was reported, it was associated with lesions in the oral cavity, paws, paw pads or nails, and could have thus contributed to some of the other systemic signs.

**Fig. 4:**
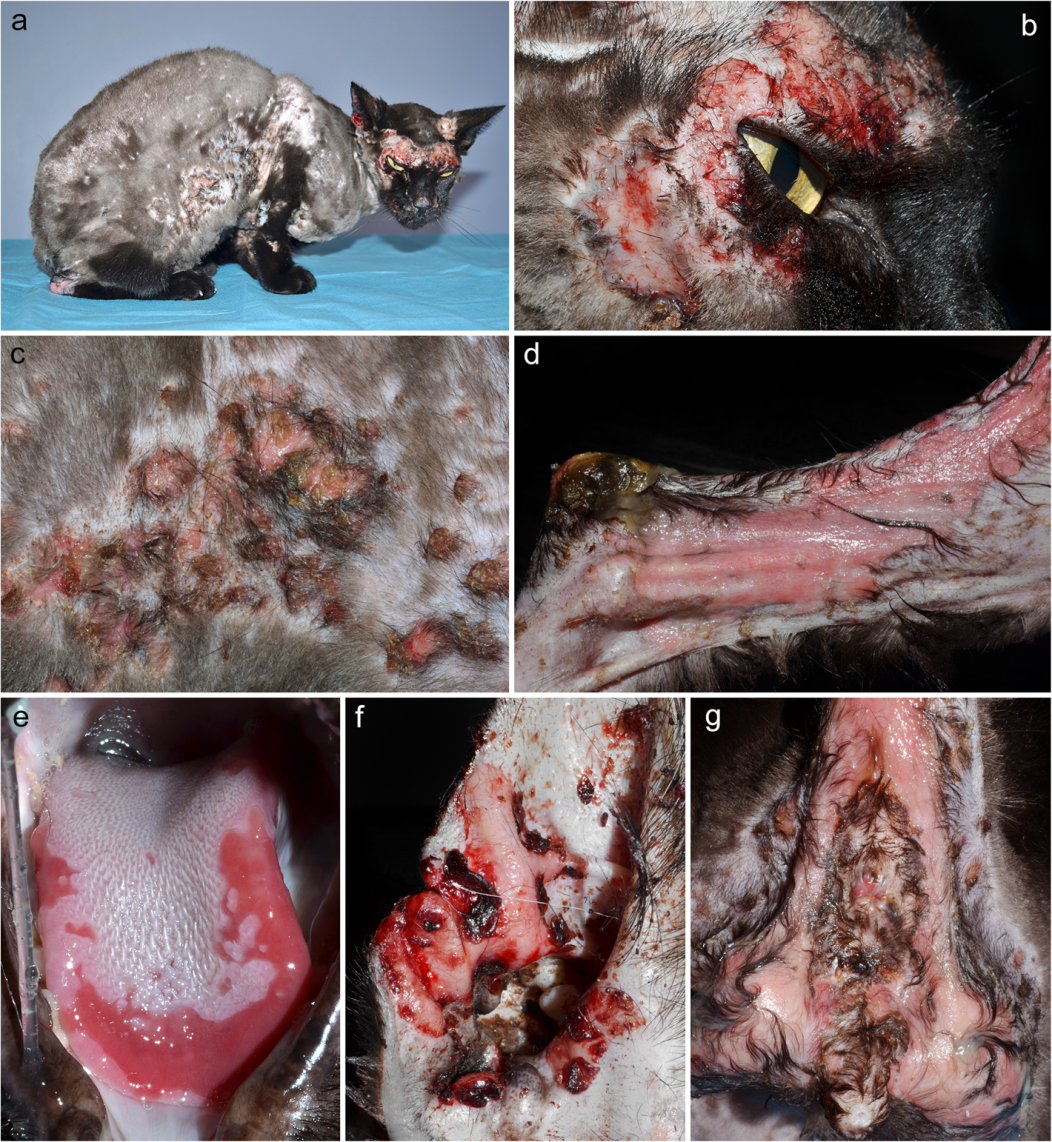
Clinical characteristics of feline pemphigus vulgaris. **a**-**d**, **f**, **g**: generalized, usually bilaterally symmetrical erosive and ulcerative dermatitis; **e**: erosive glossitis – **a**-to-**g** were taken from the same Siamese cat (courtesy of Luc Beco)

In all four cats reported with PV, skin lesions were confined to the head/face, and ulcers (Fig. 4) were the only lesion described [38]. All cats had oral cavity, lips and nasal planum involvement. In one [74], nearly the entire oral cavity was affected; it also had lymphadenopathy, halitosis and hypersalivation.

**Fig. 5:**
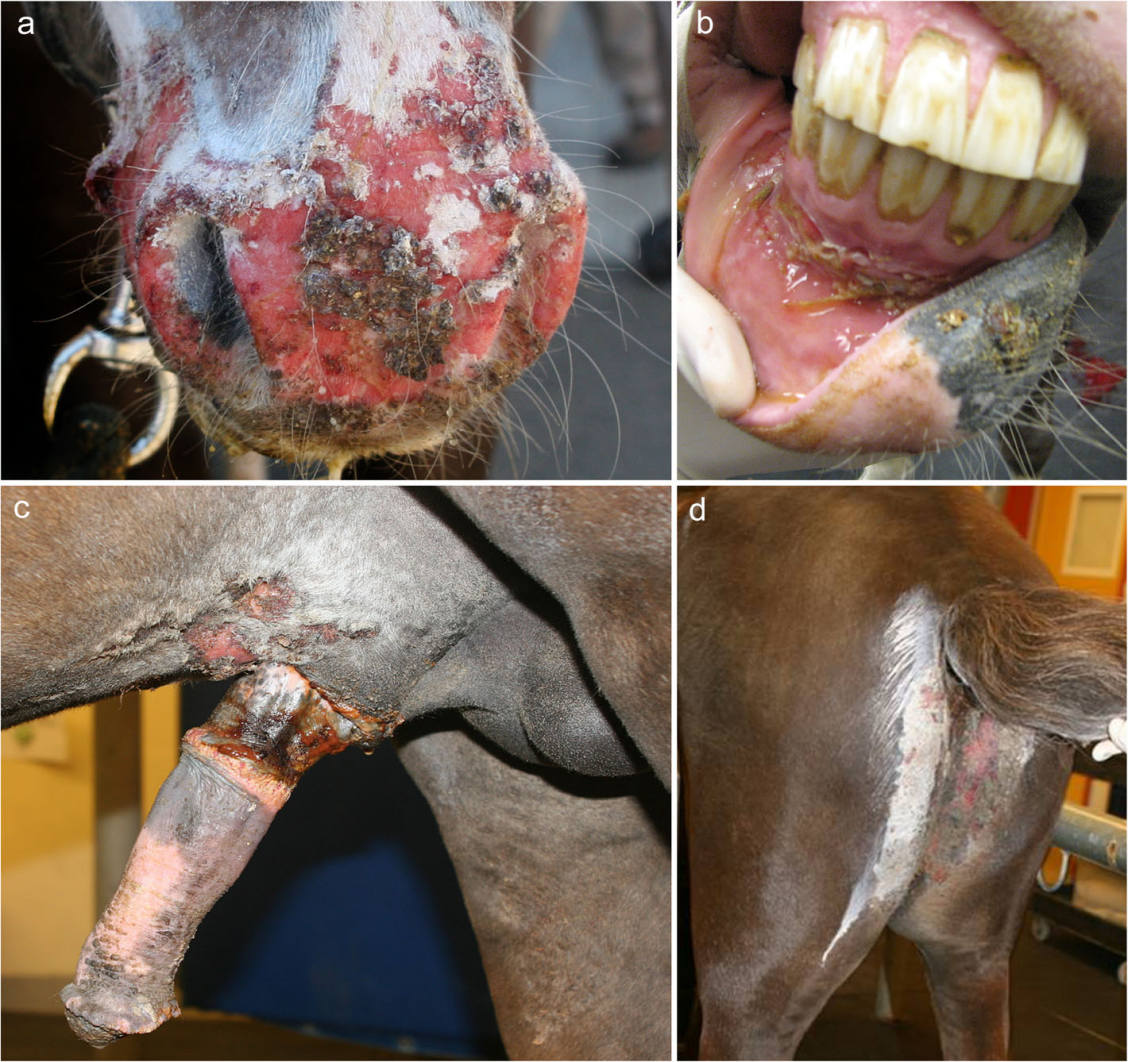
Clinical characteristics of equine pemphigus vulgaris. **a**, **c**, **f**: erosive and ulcerative dermatitis of the face, perigenital and genital area and perineum; **b**: desquamative, erosive and ulcerative gingivitis – **a**-to-**f** are from the same Welsh pony (courtesy of Stephen D White; case reported in reference [30])

The only horse with PV reported in the literature presented to the veterinarian for cutaneous lesions: crusts and scales on the tail-head [31]. The perineum (along the semimembranosus muscles), penile sheath, muzzle, mane and oral cavity were also affected (Fig. 5). In the latter, the buccal mucosa, gingiva and tongue were abnormal. The most common lesion was an ulcer, and others included erosions, crusts, alopecia (mane) and vesicles (muzzle). The lesions in the oral cavity were assumed to have been painful because the horse needed sedation for oral examination. Systemic signs were not reported. It is not known if the skin lesions developed before, concurrently, or after the oral mucosal lesions. Pruritus was reported to be confined to the muzzle.

#### Histopathology

**Fig. 6:**
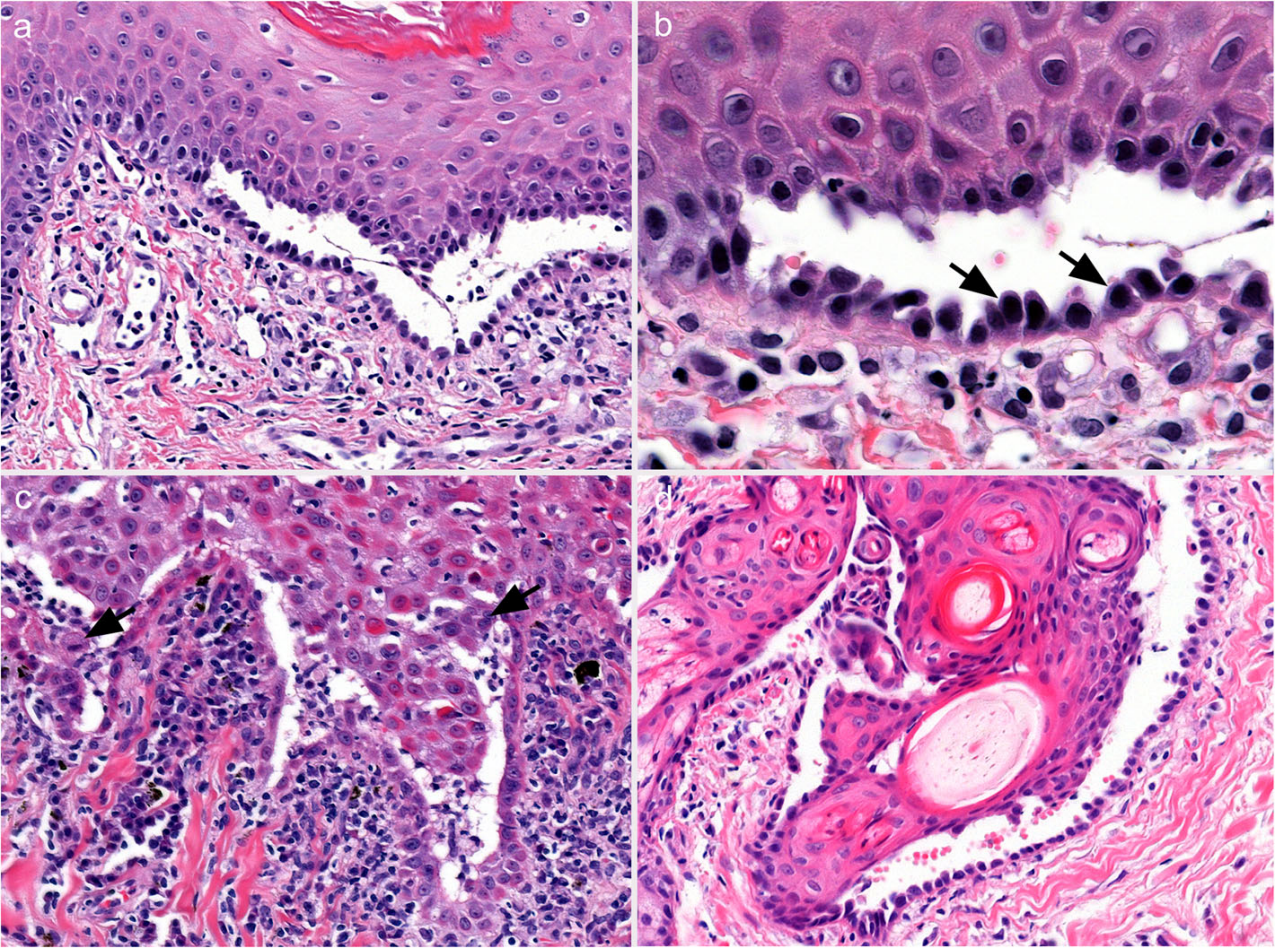
Histological characteristics of canine pemphigus vulgaris. **a** and **b**: a suprabasal cleft in the epidermis is due to keratinocyte acantholysis. Characteristically, a single row of individualized and rounded basal keratinocytes remains attached to the dermis below the cleft, seen best in image b (inset from a; arrows). Inflammation in the vesicle lumen is absent and it is minimal in the epidermis and dermis; **c**: the base of a suprabasal cleft in some patients is lined by basal keratinocytes that do not separate significantly on their sides. In the vesicle lumen, neutrophils and fewer mononuclear cells are present with a few individual and small “rafts” or clusters of acantholytic keratinocytes (arrows). Lymphocytic exocytosis in the epidermis is mild to moderate, as is dermal neutrophilic and lymphoplasmacytic inflammation. Eosinophils can occur (not shown); **d**: suprabasal clefts extend along the hair follicle infundibulum, as well as the sebaceous gland duct, and inflammation is minimal. **a**, **c** and **d** 20x - magnification. **b** – 63x magnification. Hematoxylin and eosin. All pictures are courtesy of Keith E. Linder

The key histological feature of PV in all species is suprabasal acantholysis of keratinocytes in the epidermis and/or mucosae [3, 31–33, 38, 60, 69, 81, 82]. Acantholysis leads to clefting in the epithelium just above the basal cell layer and to the formation of vesicles and/or bullae (Fig. 6). Basal cells separate on their lateral and apical surfaces and exhibit mild cellular hypertrophy, rounding, and mild basophilia. Characteristically, a single row of individualized basal cells remains attached to the basement membrane below a cleft, and is referred to as a row of “tombstones” (Fig. 6). Early suprabasal acantholysis is seen as a thin line of intercellular clear space due to cell separation just above the basal cell layer, which may be discontinuous and mimics spongiosis. Very mild keratinocyte individual cell necrosis (presumed apoptosis; controversially referred to as apoptolysis in humans [42, 83, 84]) sometimes occurs along the separation lines of small epithelial clefts (presumed early lesions) and/or the margins of formed vesicles [31] (personal observations of canine cases). The acantholysis of keratinocytes in the deep stratum spinosum occurs along the roof of the cleft, but free floating acantholytic cells, or clusters of acantholytic cells – called “rafts” – in the vesicle are typically infrequent or absent [60, 69]. In a single horse case, these free acantholytic cells were described as numerous [31]. The suprabasal acantholysis can extend to the hair follicle infundibula, including sometimes the deep external root sheath, and may involve the nail bed epithelium [60, 69, 70, 81]. The vesicles contain clear serous fluid and neither leukocytes, fibrin, nor hemorrhage accumulate in high amount in the vesicles. A mild exocytosis of lymphocytes and/or neutrophils occurs in the epidermis and mucosal epithelium. A perivascular-to-interstitial pattern of inflammation predominates in the dermis and submucosae and includes neutrophils, lymphocytes, and plasma cells. Eosinophils are present in some cases in the dog, which might be more often seen in skin than in mucosa lesions [81]. A band-like (lichenoid) inflammatory cell infiltrate may occur just below the epithelium, which is most often in the mucosal, perimucosal, and nasal planum areas, where it is considered a stereotypic tissue response rather than a diagnostically-specific inflammatory pattern [60, 69, 81, 82]. The vesicles and bullae often rupture to produce deep erosions. Importantly, microscopically-diagnostic areas of suprabasal acantholysis, observed as single row of attached, rounded, and separated basal cells, can often still be found at erosion margins and along the floor of recently ruptured vesicles. It should be noted that deep erosions from other causes can be lined by a single layer of epithelial cells, but these cells are usually flattened and elongated, and thus more typical of a wound healing response. Older lesions ulcerate centrally and these areas are not diagnostic. In early, uncomplicated, eroded or ulcerated areas, the stromal surface architecture of the dermis or submucosa is typically retained, which is attributed to an intact basement membrane. Scarring is usually not seen in deep pemphigus lesions. Secondary bacterial infection can promote additional histological lesions, which can complicate and degrade PV-specific histopathologic changes.

#### Treatment and outcome

In 2015, the first guidelines for the treatment and management of human pemphigus, including PV, was published [3]. These guidelines were based on the consensus of a group of European dermatologists with expertise in human pemphigus. In 2020, these guidelines were updated by an international panel of experts [4]. The first-line therapy for human PV is oral or intravenous (IV) predniso(lo)ne alone, or in combination with adjunctive immunosuppressants. In the 2020 guidelines, injections of the anti-CD20 monoclonal antibody rituximab were included as a first-line therapy for moderate-to-severe pemphigus and/or for patients without disease control despite treatment with systemic glucocorticoids (GC) and immunosuppressive agents [4]. The first-line adjunctive steroid-sparing immunosuppressant agents used most commonly are azathioprine (AZA) and mycophenolate mofetil (MMF). Supportive treatments that might be recommended are a proper dental care, intralesional injections of GC (e.g., triamcinolone acetonide) for isolated lesions, potent topical GC (e.g., clobetasol propionate), or topical calcineurin inhibitors.

**Fig. 7:**
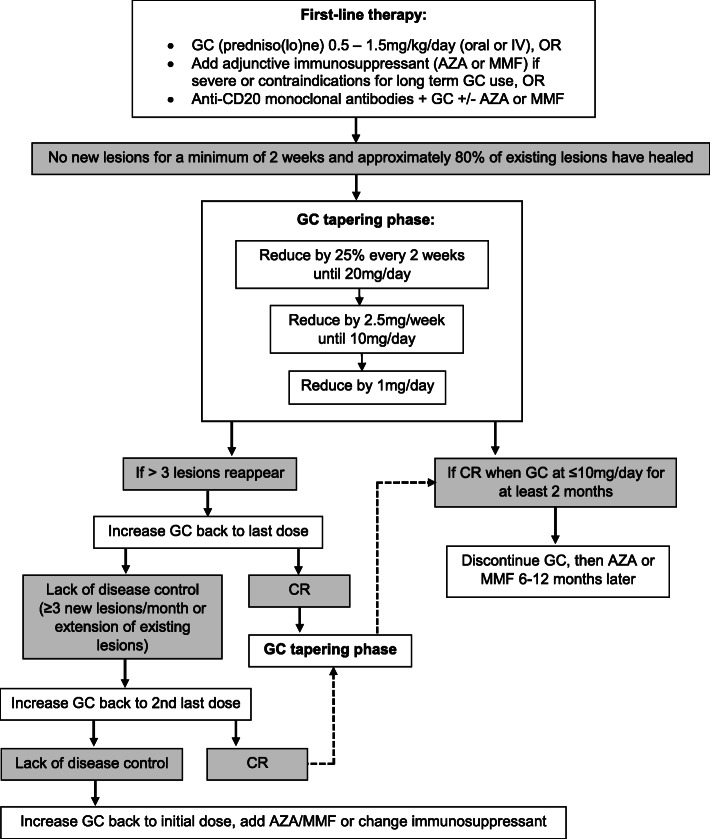
Flow diagram of the treatment approach to human pemphigus. This was adapted from Murrell et al. J Am Acad Dermatol 2020 (reference [4])

The treatment approach for human pemphigus—including PV—recommended in the 2020 guidelines is summarized in Fig. 7. The complete remission (CR) (i.e., the full healing of all lesions) is expected to take 1 to 3 months [4]. There is limited evidence that the addition of adjunctive immunosuppressants will result in a better outcome compared to GC monotherapy alone, and there are no studies that have shown that intravenous GC-pulse therapy is superior to conventional first-line therapy with oral GC with or without immunosuppressants [4]. However, GC-pulse therapy in addition to a conventional regimen is suggested for refractory cases. The discontinuation of systemic GCs can occur when the CR is achieved on a minimal therapy of predniso(lo)ne at 10 mg or less per day, and adjuvant immunosuppressive drugs can be stopped 6 to 12 months after obtaining the CR of lesions.

In dogs with PV, the information on disease treatment and outcome can be inferred from 22 reports [11, 26–28, 38, 46, 50, 55–60, 62, 64–67, 69–71] including 47 dogs. The final outcome was not stated for the last seven [38, 46, 60, 63, 68, 70]. Overall, a CR was obtained in 26/40 dogs (65%) that received treatment. The time-to-CR varied between 2 and 36 weeks. A partial remission (PR) was reported in 12 dogs [46, 55, 57–60, 67, 71], although the definition of PR varied between cases. One dog [61] died before treatment could be initiated. Fifteen dogs were euthanized for the following reasons: an adverse reaction to therapy, including immunosuppression (6/15 dogs; 40%) [38, 46, 55, 59, 60], a lack of response to treatment (7/15 dogs; 47%) [46, 56, 67, 69, 71], a reluctance to maintain the dog on treatment (1/15 dogs; 7%) [26], and finally, one dog (7%) was euthanized without being treated [69]. Three of the dogs that were euthanized [67, 69] had had a CR of lesions at some point in the course of their disease. A spontaneous remission without any treatment was only reported in 1/40 dogs (3%) [38]. In 26 dogs in which a disease CR was achieved and follow-up information was available, 10 (38%) had had a relapse: clinical signs flared in five of these dogs (50%) when the dose of ciclosporin (CsA) (three dogs) [67] or GC (two dogs) [27, 66] was tapered, and in three dogs when one or all treatments were discontinued [58, 69]. In two dogs, lesions relapsed while they were still on treatment (GC [65] or heparin monotherapy [64]) that had been successful in inducing CR.

Treatment regimens varied widely, and they included the following (in descending order of frequency): GC, AZA, CsA, cyclophosphamide (CYC), chlorambucil, aurothioglucose, heparin and doxycycline. At the time of CR, 50% of the dogs (13/26) were receiving a systemic GC monotherapy [27, 38, 66, 69, 70]. A combination therapy of systemic GC with a single adjunctive immunosuppressive/immunomodulatory drug resulted in CR in 5/26 (19%) dogs [11, 28, 50, 58, 62]; of these, the most common drug used was AZA (3/5; 60%) [11, 50, 62]. One dog treated with a combination of prednisone and doxycycline [58] experienced a relapse of the disease after discontinuation of the doxycycline; the CR was achieved again when doxycycline was restarted. In three dogs [67], a CR was obtained with CsA alone. In one dog, CR was achieved with heparin monotherapy after the previous administration of GC and chlorambucil had failed to provide benefit [64].

An oral GC (prednisolone) monotherapy resulted in a CR in 3/4 cats (75%), while it was obtained with aurothioglucose monotherapy in the remaining cat [38, 73]. The follow-up period ranged from 2 to 5 years, during which relapses were not reported in any of these cats.

In the single case report of equine PV [31], treatment with systemic (intramuscular dexamethasone, followed by oral prednisolone) and topical (0.015% triamcinolone acetonide) GC initially resulted in an improvement of skin lesions, but over the following months, the horse developed laminitis of all legs, bilateral corneal ulcers, and the skin and oral lesions became refractory to GC therapy. The sequential addition of azathioprine, aurothioglucose, and dapsone failed to induce CR, and the horse was finally euthanized 4 months after the initial presentation.

#### Implications for practice

Most cases of canine PV exhibit a mucocutaneous phenotype with nearly all having oral involvement, and erosions/ulcers being the most common lesions. The differential diagnoses for dogs presented for oral erosions/ulcers include (but are not limited to) mucous membrane pemphigoid, epidermolysis bullosa acquisita, erythema multiforme (EM) major, oral variants of epitheliotropic T-cell lymphoma [85], severe periodontal disease, and, rarely, PNP. The definitive diagnosis requires a biopsy and the histopathological confirmation of suprabasal acantholysis. The importance of selecting the best lesion(s) to biopsy cannot be overemphasized. Biopsies collected from the centers of old erosions and ulcers are unlikely to retain the basal keratinocytes critical for making the diagnosis. For dogs, cats or horses, the biopsy of intact vesicle(s) is ideal but they are far less common compared to erosions/ulcers. Biopsying the latter should include one-third of the erosion/ulcer and two-thirds of the perilesional skin/mucosa, as recommended by the 2015 European guidelines for human pemphigus [3]. Multiple biopsies are essential to capture diagnostic areas within secondary erosions and ulcers and at least 5 to 6 biopsies should ideally be collected.

Once a definitive diagnosis of PV is achieved, treatment should begin immediately with high dose GC, either as monotherapy (at least 3 mg/kg/day of predniso(lo)ne for dogs and up to 3 mg/kg twice daily of prednisolone for cats) or with an adjunct immunosuppressant; if the latter option is selected, a slightly lower dose of GC could be used. Based on the published reports in canine PV reviewed herein, high-dose GC monotherapy has the highest probability of success in inducing a CR, but this evidence is debatable, as the number of dogs with reported treatment outcome is too small for strong conclusions to be made. The addition of adjunctive immunosuppressant should also be considered if the patient has a contraindication for long-term GC therapy (i.e., diabetes mellitus, gastric ulcer, etc.) or develops severe adverse effects from it. Among immunosuppressants, AZA is a reasonable option because it was the most common single drug that resulted in disease CR when combined with GC. Finally, CsA, either as monotherapy or in combination with oral GC, may be attempted should there be contraindication(s) for AZA (i.e., hepatic injury and/or myelosuppression). In the single case report in which CsA monotherapy was prescribed, the following dose regimen was used: 25 mg/kg once daily for 1 week, followed by weekly reduction by 5 mg/kg until a minimum dose of 10 mg/kg once daily [67]. Because CsA inhibits down-stream signaling of pro-inflammatory cytokines associated with the activation of T cells, one might expect a slower onset of clinical activity, and, as such, the concurrent short-term use of oral GC may accelerate the clinical response [86].

Animal PV, as its human homologue, is a chronic disease, and relapses can be expected. The two most common reasons dogs with PV were euthanized were because of adverse drug effects or a lack of response to therapy. It is therefore very important for practitioners to discuss the treatment plan and prognosis with the owners before therapy is commenced.

Finally, PV affecting only the nails/claws cannot be clinically distinguished from canine symmetric lupoid onychodystrophy (SLO) or idiopathic onychitis/onychomadesis, unless there is erosion/ulcer on the periungual region [60], which is rarely seen in canine SLO. The decision to biopsy these lesions can be difficult to make because it either requires digital amputation or onychobiopsy without onychectomy [87], with the latter method requiring practice and experience to execute. With canine PV being much rarer than SLO, especially the nail-restricted-PV-variant, it is reasonable that the first-line therapy should be targeted for SLO, and if there is lack of response, biopsy may be considered. If skin or mucosal lesions are available to biopsy, then biopsies of claw lesions are not needed. If the lesions are restricted to the claw, then an entire affected claw should be collected by amputation of the distal phalanx, ideally of an affected dewclaw to minimize clinical impact.

#### Implications for research

In veterinary medicine, histopathology remains the only reliable method to diagnose PV. Unfortunately, animals with PV lesions confined to the tongue and/or palate can be challenging to biopsy, which requires general anesthesia. Therefore, the availability of a less invasive or ‘traumatic’ diagnostic test would be beneficial, especially in patients in which a general anesthesia poses a high risk (e.g., congestive heart failure, chronic kidney disease, etc.). In 2008, Nishifuji and colleagues reported on the development of an enzyme-linked immunosorbent assay (ELISA) for the detection of circulating IgG AA against DSG3 in dogs with pemphigus [88]. In that study, the titer of canine DSG3 AA was significantly higher in dogs with PV compared to normal dogs, and the authors concluded that a canine DSG3 ELISA might be a valuable screening tool for the diagnosis of canine PV. However, that study only involved sera from six dogs with PV and, therefore, future studies with a larger number of samples would provide more information on the sensitivity and specificity of this diagnostic tool. This could then potentially lead to the study of the correlation of circulating anti-DSG3 AA with the severity of the disease, allowing practitioners to know when would be the optimal time to taper the treatment dosage(s).

Rituximab (RTX) is the first monoclonal antibody (MAB) approved for the treatment of CD20 antigen-positive, B-cell non-Hodgkin’s lymphoma in humans [89]. Since its approval by the Food and Drug Administration (FDA) in 1997, RTX has also been used for the treatment of antibody-mediated autoimmune diseases such as systemic lupus erythematosus [89], rheumatoid arthritis [89] and, more recently, as a first-line therapy for human PV [4]. In veterinary medicine, a novel anti-canine CD20 MAB was successfully developed in 2015 [90], with another study identifying a candidate therapeutic antibody for the treatment of canine B-cell lymphoma [91]. Since canine PV appears to have a similar pathogenesis as its human counterpart, future studies to investigate the potential of anti-canine CD20- or another anti-B cell- MAB for the treatment of canine PV should be investigated.

### Pemphigus vegetans

#### Introduction

In humans, PVeg is a rare clinical variant of PV [92] that tends to affect the periorificial regions or large skin fold areas [3]. The skin lesions, however, are different from those of PV. Rather than erosions/ulcers, papillomatous vegetations and/or pustular skin lesions are distinct clinical features of PVeg [3]. Two forms of PVeg have been described: the Neumann- and the Hallopeau types [3, 92]; the clinical features of both of these forms in humans will be discussed below. In humans, the diagnosis of PVeg is based on skin lesions, histopathology (similar to PV with additional features including papillomatosis and hyperkeratosis [34, 93]), and either a positive DIF or the serological detection of AA against epithelial cell surface antigens [3].

#### Historical perspective

Pemphigus vegetans was first described by the Austrian dermatologist Isidor Neumann in 1886 [23] – the clinical features included bullae that would rupture and develop into “dense warty granulation” that spread serpiginously (i.e., in a snake-like pattern). More than a decade later, Henri Hallopeau reported five patients that exhibited polycyclic eruptions of pustules that rupture and formed “firm pink papillomas”. Together with a similar case also reported by himself 9 years previously, Hallopeau named this variant of PVeg as “pyodermite végétante” in 1889 [94]. It was not until 1965 that Lever classified Hallopeau’s “pyodermite végétante” as PVeg Hallopeau-type in his book [25]; this name has been used in the medical literature until today.

The first canine PVeg was reported in 1977 by Scott [95]. Three years later, Schultz and Goldschmidt reported PVeg in a 7-year-old chow-chow [17]. As the clinical and histopathological features in this dog do not fit those of PVeg, but rather resemble those of canine PF, this report of canine “PVeg” will not be discussed further. Three decades after Scott’s report, Heimann and colleagues reported a second dog with lesions resembling those of human PVeg [96]. The most recent case of this disease was reported in 2012 by Vercelli and Cornegliani [97].

#### Incidence and prevalence

Pemphigus vegetans accounts for 1 to 2% of all cases of human pemphigus [54]. In one study from Tunisia [92], the incidence of PVeg was 0.58 case/year with a prevalence, in a single hospital, of 0.084% of patients. In dogs, PVeg is extremely rare with only three cases reported; there is thus insufficient information to estimate its incidence and prevalence. Pemphigus vegetans has not been reported in other animal species.

#### Etiopathogenesis

Pemphigus vegetans is considered a variant of PV, and, therefore, the etiopathogenesis of PVeg is believed to be similar to that of PV. The very few immunologic studies of human PVeg indicated that the antigen most commonly recognized by AA was the 130-kDa PV antigen DSG3 [98–101]. In one recent study involving 17 patients [92], the most common immunoreagents seen deposited in DIF were IgG and complement C3, and a commercial ELISA performed in eight patients showed IgG AA recognizing DSG3 in all eight patients, with two patients also positive for anti-DSG1 AA. The vegetative skin lesions in the intertriginous area are thought to due to occlusion and maceration with a subsequent bacterial infection, and/or an associated Th2-mediated immune reaction that involves both IgG AA and/or cytokines that promote an epithelial proliferation and eosinophil chemotaxis [92].

Immunologic investigations were only performed in one dog with PVeg [96]. In this case report, DIF showed the intercellular deposition of IgG in all epidermal layers, with the strongest fluorescence in the stratum spinosum. Additionally, circulating IgG AA were shown to recognize DSG1, but not DSG3.

#### Signalment

Most of the literature on human PVeg is written as subtopics of PV, and we thus assumed that either the signalment of human PVeg is similar to that of PV, or the rarity of PVeg in humans and/or articles devoted to PVeg alone does not allow for the generation of meaningful signalment data. In one study, the median age of onset was cited as 48 years (range: 24 to 78 years) with a female-to-male ratio of 4.7 [92]; these data are somewhat similar to those of other patients with PV.

The three dogs with PVeg were a wirehaired fox terrier [95], a greater Swiss mountain dog [96] and a bulldog [97]. There was one female and two male dogs, and the age of onset of skin lesions varied between 1.5 and 9 years.

#### Clinical signs

Human PVeg is classified into Neumann and Hallopeau types [3]. The Neumann-type of PVeg usually begins with vesicles and blisters that rupture and then form hypertrophic vegetative plaques, whereas an Hallopeau-type should be considered when the skin lesions begin with pustules and evolve into hyperkeratotic verrucous papillomatous vegetations [54, 92, 93]. These skin lesions tend to develop at periorificial regions (e.g., the vermilion border of the lips) or intertriginous areas (i.e., large skin folds such as axillary, inguinal, perianal, mammary folds) [3, 34, 54]. Similarly to PV, an oral cavity involvement is common [54, 92], and it is often the first site affected [93]; oral vegetations are rare, however [93]. Other affected regions include the scalp [54] and nails/fingers [102, 103]. Although pruritus is not a common feature in human PVeg, it was reported in 5/17 patients (30%) in one study [92].

**Fig. 8:**
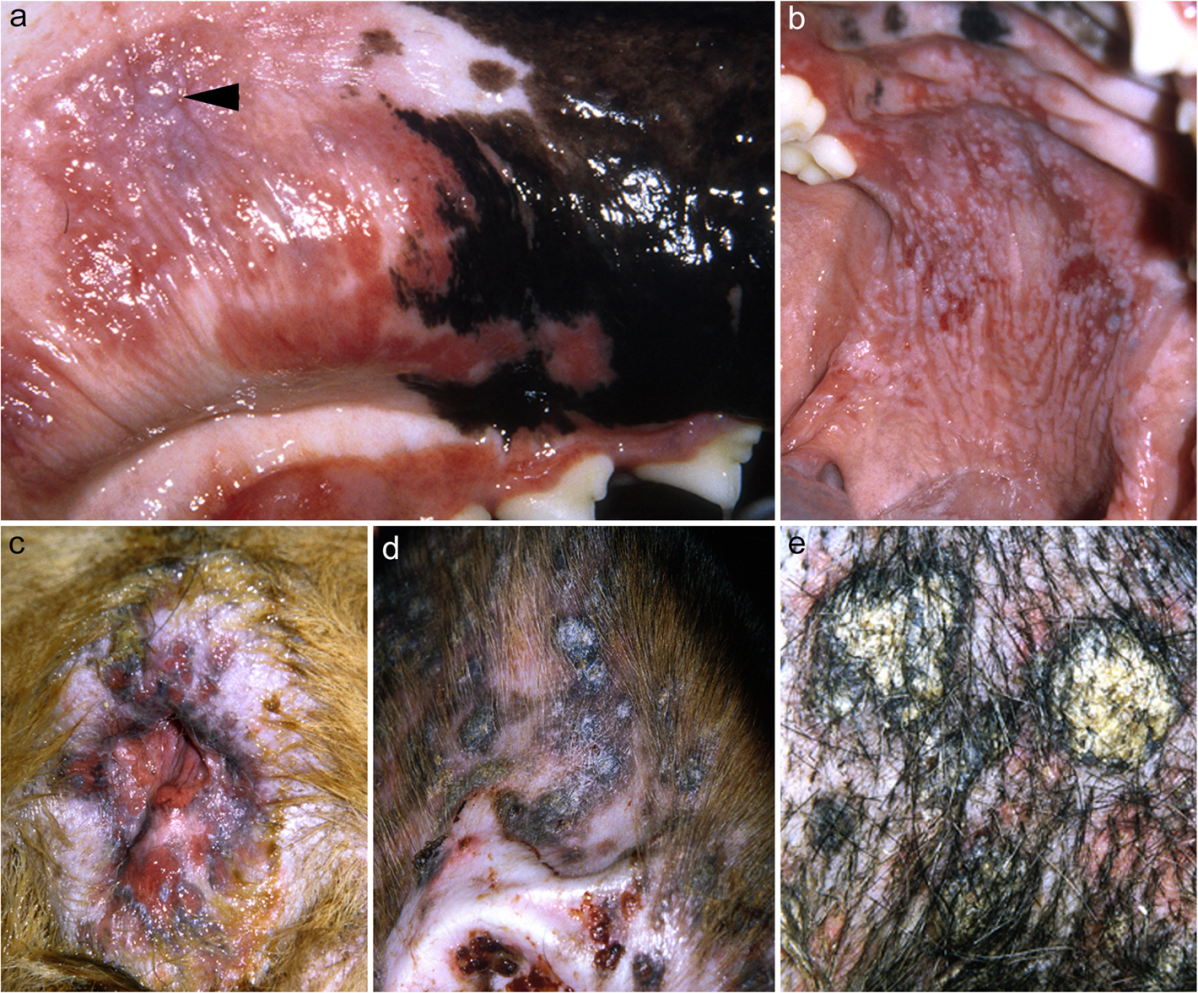
Clinical characteristics of canine pemphigus vegetans. **a**: erosive and desquamative stomatitis with sloughing of the gingival epithelium (arrowhead); **b**: erosive and ulcerative dermatitis affecting the palate; **c**: erosive and ulcerative proctitis with perilesional hyperpigmentation; **d**: coalescing, hyperpigmented, scaly and crusted papules on the concave pinna; e: wart-like scaly papules on the sternum – **a**-to-**e** were taken from the same greater Swiss mountain dog (courtesy of Luc Beco; case reported in reference [96])

In the first case of canine PVeg, the dog was presented to the veterinarian with a 5-year history of skin disease [95]. Initial skin lesions consisted of pruritic pustules that developed on the axillae, chest, abdomen, and groin. In the remaining two cases [96, 97], both dogs presented to the veterinarian with a primary complaint of crusts and pruritus, with alopecia reported in the last dog [97]. In these two dogs, the skin lesions first developed on the head and neck. In all three dogs, the skin lesions eventually progressed to a generalized distribution. A mucosal and mucocutaneous involvement was only reported in one dog [96]: the affected regions were the oral cavity, lips, prepuce and anus (Fig. 8). In the oral cavity, the palate and gingiva were affected. The dogs with PVeg reported by Scott [95] and Vercelli [97] had only cutaneous involvement on the head [97], trunk [95, 97] and limbs [95, 97], and there were no systemic signs or pain. Interestingly, in the dog with PVeg reported by Vercelli [97], the pruritus decreased after 2 months of systemic and topical antimicrobial therapy, along with an elimination diet trial with a hydrolyzed diet. Therefore, it is possible that the pruritus reported in this dog could either be associated with a secondary skin infection (which was thought as one of the possible causes of vegetative skin lesions in human PVeg [92]), a cutaneous adverse food reaction, or the combination thereof.

As the disease progressed, exophytic/hyperplastic verrucous crusts, erosions and/or ulcers were reported in all three dogs while, pustules and/or vesicles were only visible in two of them [95, 96]. Pustular lesions were distributed on the axillae [95], chest [95], abdomen [95, 96], groin [95, 96], proximal limbs [95] and paws [96]; vesicles were also reported on the gingiva in one dog [96].

In the first report of canine PVeg [95], the progression of skin lesions (pustules that evolved to verrucous vegetations) resembles that of the Hallopeau phenotype. Unfortunately, in the remaining two case reports [96, 97], it was not stated if the skin lesions began with pustules or vesicles and therefore, we could not ascertain if their PVeg was of Neumann- or Hallopeau type. However, the dog in Heimann’s report [96] exhibited a combination of Neumann- and Hallopeau phenotypes, as was reported in a recent retrospective study of human PVeg in 2011 [92].

#### Histopathology

**Fig. 9:**
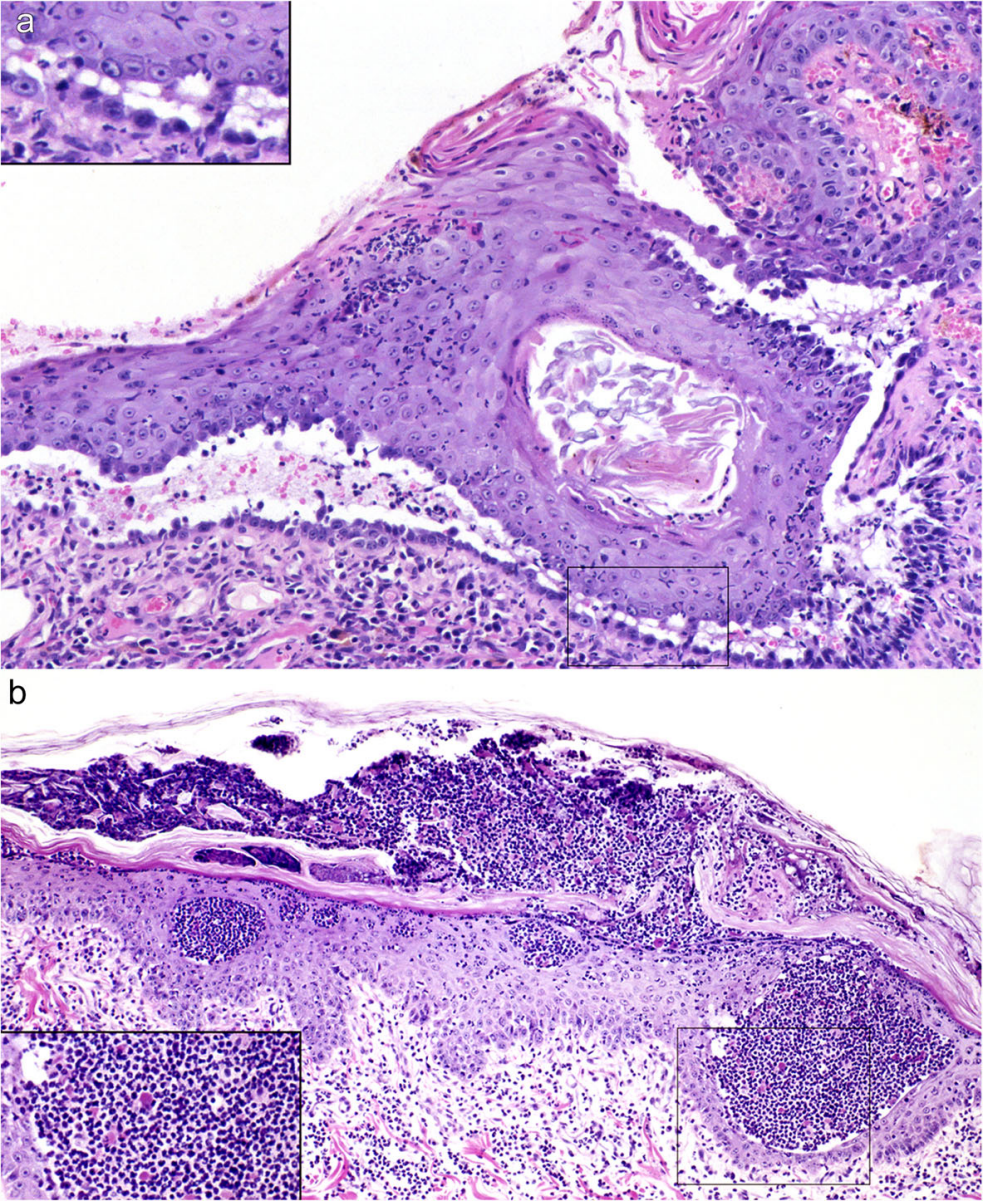
Histological characteristics of canine pemphigus vegetans. **a**: an acantholytic suprabasal cleft, typical of pemphigus vulgaris, is present in the prepucial skin and is associated with a single row of attached basal cells at its base (inset). Neutrophilic exocytosis and small pustule formation are present more superficially in the epidermis. Mixed neutrophilic and lymphoplasmacytic inflammation is in the submucosa; **b**: Multiple pemphigus foliaceus-type neutrophilic acantholytic pustules (inset) are present in the superficial and deep epidermis of the abdomen. Hematoxylin and eosin (case reported in reference [96]). All pictures are courtesy of Keith E. Linder

Because so few cases of PVeg are described in animals, the features of the human disease are used to identify animal cases. The salient histopathological features of PVeg in humans are distinctly hyperplastic epidermal lesions and a suprabasal acantholysis typical of PV (see above), the latter of which is essential to confirm the diagnosis [3, 92]. This is because PVeg is classified as a subset of PV and because deep epidermal pustules also occur with animal PF—especially in dogs—and PF should not be mistaken for one of the deeper pemphigus variants [2]. However, PV-type suprabasal acantholysis might not be present in all biopsies or it could have disappeared in some chronic lesions of PVeg in humans, and thus multiple skin biopsies and a compatible clinical diagnosis might be required in some cases in animals [92, 95, 104]. An irregular to papillated epidermal hyperplasia, also described as verrucous or (pseudo)-epitheliomatous, is typical of PVeg, and hyperkeratosis is also a feature of some chronic lesions. Mucosal lesions, when present, are less hyperplastic than cutaneous ones, but they feature a PV-type acantholysis. Intraepidermal pustules with acantholysis can also be present concurrently, but they are not a diagnostic criterion for PVeg [3]. Inflammation is typically eosinophilic and neutrophilic. Of the three cases in dogs reviewed herein, the microscopic description of the case by Heimann and colleagues is the most detailed and most resembles the human disease homologue (Fig. 9) [96]. In this dog’s skin and oral mucosa, suprabasal acantholysis was typical of PV; suprabasal clefts were bordered by a single row of rounded and individualized basal keratinocytes below, and they contained scant neutrophils, mononuclear cells, and erythrocytes, as well as rare acantholytic keratinocytes. In addition, intraepidermal pustules occurred in the superficial and deep epidermis and contained intact neutrophils, eosinophils, and acantholytic keratinocytes. Both suprabasal acantholysis and intraepidermal pustules were found in hair follicle infundibula. In the skin, all biopsies exhibited epidermal hyperplasia and orthokeratotic hyperkeratosis, which was papillated and exophytic in the more fully-developed lesions. A perivascular-to-interstitial pattern of superficial dermal inflammation contained neutrophils, lymphocytes, macrophages/dendritic cells, mast cells, and rare eosinophils. Fewer histological features were described in the case by Vercelli and colleagues, and the evidence for a diagnosis of PVeg was more limited [97]. The skin lesions used for this diagnosis were hyperplastic and contained suprabasal acantholysis reminiscent of PV that affected the hair follicle external root sheath. Epidermal acantholysis was not demonstrated, and lesions did not occur in the oral cavity. Other features of this case included acrochordon-type fibroepithelial hyperplasia, follicular orthokeratotic hyperkeratosis, and an uncharacterized dermatitis; these changes were partially attributed to pyoderma, yeast-overgrowth, and possibly allergic skin disease. In the first case reported by Scott [95], the diagnosis of PVeg was made by supportive clinical signs and the presence of suprabasal-to-subcorneal neutrophilic and eosinophilic micro-abscesses with acantholytic keratinocytes [95]. These changes, unfortunately, also can be found in some dogs with PF.

#### Treatment and outcome

Since PVeg is considered a rare variant of PV, the treatment guidelines for it are similar to those of PV [4]. Most of the articles on human PVeg contain single case reports, and, therefore, it is difficult to generate information that would give an accurate and meaningful overview on the treatment and outcome of this rare disease. Nevertheless, the Hallopeau-type PVeg is thought to have a better prognosis than the Neumann-type, because the lesions in the latter are more severe [54, 92, 93]. One study reported a high relapse rate with the mean duration of CR before a relapse of 23 months [92]. This study also did not find any statistically significant difference between the two subtypes of PVeg in relation to the relapse rate, wound healing period, survival, or death.

In the first report of canine PVeg, the dog was treated with prednisolone monotherapy, and the CR of signs was achieved after 3 weeks [95]; the follow-up period was 5 months. The remaining two cases of canine PVeg were treated with the combination therapy of predniso(lo)ne and AZA, with the CR of signs was obtained in one dog [96] and a PR in the other [97]; the follow-up period lasted 3 years and 10 months, respectively. As in human PVeg, relapses of skin lesions were reported in all three dogs. Two dogs [96, 97] died due to causes unrelated to PVeg or adverse effects of immunosuppression.

#### Implications for practice

The diagnosis of PVeg in dogs can be a challenge because the pruritic and vegetative (that is, proliferative, exophytic or wart-like) crusts or plaques can be mistaken as those of a superficial pyoderma. Indeed, in humans, atypical PVeg affecting the scalp or fingers only have been mistaken as sebopsoriasis [105] or psoriatic acrodermatitis [103], respectively. When presented with dogs with vegetative crusts that initially developed on the head and neck, a thorough dermatological examination, including the oral cavity, should be performed to detect the presence of other lesions such as vesicles, erosions and/or ulcers. A skin cytology, bacterial culture with susceptibility testing, and response to antimicrobial therapy should be performed to rule out a superficial pyoderma. If the skin lesions then persist, numerous skin biopsies should be collected, ideally five-to-six, with additional ones from any oral cavity lesions, if present. Because erosions and/or ulcers are often found underneath the vegetative crusts, skin biopsies should also include perilesional skin to increase the chances of capturing a suprabasal acantholysis. As with PV, systemic GC with or without adjunctive immunosuppressants should be considered the mainstay of therapy and should begin immediately once the diagnosis is confirmed. Pet owners should be informed that canine PVeg is a chronic relapsing autoimmune skin disease that likely requires a prolonged immunosuppression.

#### Implications for research

The extreme rarity of PVeg in dogs is a hurdle for research on the etiopathogenesis, diagnostic methods, and new interventions. A multi-institutional study should globally engage veterinarians having diagnosed a dog with PVeg to assemble a large number of cases, and, thus, shed more light on the epidemiology, clinical phenotypes, and treatment outcomes of this disease. This could then form the basis for more specific studies such as investigating inflammatory pathways and/or cytokines that might be involved in the development of vegetative lesions, and possibly newer therapy with less immunosuppressive effects targeting specific pathway(s). Finally, it would be interesting to investigate why suprabasal acantholysis developed in the dog that did not have circulating AA against DSG3 [96].

### Paraneoplastic pemphigus

#### Introduction

The adjective “paraneoplastic” refers to syndromes that are associated with malignant tumors, but that are unrelated to their invasiveness or metastases [106, 107]. The term “paraneoplastic” is derived from the Greek roots *para*, meaning “to, at, or from the side of”, *neo*, meaning “new” and *plasma*, meaning “formation” [106] – this term was introduced by Guichard and Vignon in 1949 (cited in [107]). Cutaneous paraneoplastic syndrome (CPS) refers to cutaneous abnormalities seen in patients with malignant diseases that are not directly associated with the malignant disease itself. In humans, the malignant diseases that lead to a CPS can be in different systems, such as the alimentary (i.e., necrolytic migratory erythema), hematolymphoid (i.e., paraneoplastic pemphigus) and aerodigestive (i.e., Bazex syndrome) systems [106].

In this section, we will focus on PNP, a CPS that is a deep form of pemphigus, and where relevant comparisons with the human counterpart are made.

#### Historical perspective

The term “paraneoplastic pemphigus” was first introduced to the medical literature by Anhalt and colleagues in 1990 [108]. In his paper, Anhalt reported five patients with an underlying malignant neoplasm that developed vesicles and/or erosions in the oral cavity, mucocutaneous junctions, and/or skin. Four of five patients’ skin biopsies showed suprabasal acantholysis (similar to PV) with necrosis (apoptosis) of individual keratinocytes that resembled that seen in EM; these four patients eventually died due to their malignant neoplastic diseases.

In veterinary medicine, Stannard and colleagues reported an 11-year-old spayed female Boxer dog with ulcerative glossitis and cheilitis, poor body condition and lethargy [27]. Although skin biopsies revealed suprabasal acantholysis, which made the authors report this dog has having PV, published photomicrographs show a lymphocytic satellitosis of epidermal keratinocytes, at multiple epidermal levels, with their nuclei having condensed chromatin suggestive of apoptosis. The co-occurrence of PV and EM histological lesions, coupled with the presence of an intrathoracic thymoma diagnosed via necropsy, make us logically postulate that this dog actually had PNP rather than PV. Since then, two more case reports of canine PNP have been published in 1998 [109] and 2005 [110]. Paraneoplastic pemphigus has been reported also in one cat [111], but not in any other animal species. There is an additional case report of a 6-year-old Tennessee walking horse gelding that developed a bullous stomatitis that resolved when a localized hemangiosarcoma was surgically removed [112]. Although the outcome of this horse suggests a form of CPS, the histopathological features of mucosal lesions were not consistent with those of PNP, and this case therefore will not be discussed in this review.

#### Incidence and prevalence

Paraneoplastic pemphigus is a rare autoimmune mucocutaneous disease with less than 500 cases reported in the human medical literature [113]. It accounts for 3 to 5% of all pemphigus cases [114]. In one study, hematologic malignancies accounted for 84% of PNP, with non-Hodgkin’s lymphoma being the most common disease (38%) [115]. In another study involving 17 patients with PNP, 13 (76%) had Castleman’s disease—a rare lymphoproliferative disorder that behaves like a lymphoma—two (12%) had a mediastinal thymoma, and one each had a non-Hodgkin’s lymphoma or a follicular dendritic cell sarcoma [116].

There are only four PNP cases reported in domestic animals (three dogs and one cat) and therefore, there is insufficient information to estimate the incidence and prevalence of this exceedingly rare disease in these species. The malignancies associated with the three dogs were a thymoma [27], a thymic lymphoma [109] and a splenic sarcoma [110], whereas the single cat with PNP had a lymphocytic thymoma [111].

#### Etiopathogenesis

The pathogenic IgG AA in PNP are polyclonal and target a wide array of keratinocyte-derived proteins such as DSG3, DSG1, DSC1, DSC2 and DSC3, plakin family proteins (envoplakin, periplakin, desmoplakin 1 and 2, plectin and BP230) and the alpha-2-macroglobulin-like-1 protease inhibitor [117–119]. The plakin family proteins envoplakin and periplakin, are most consistently targeted by PNP AA [113]. In fact, indirect IF using bladder epithelium (which is rich in plakins) is a highly specific (98%) method to differentiate PNP from other forms of pemphigus (cited in [113]). The possible mechanisms leading to autoimmunity in PNP include the tumor-induced production of AA, the cross-reactivity of tumor and epithelial antigens, as well as epitope spreading [117]. The human leukocyte antigen (HLA)-CW*14 and HLA-DRB1*03 seem to be associated with the development of PNP [113].

Immunopathological studies were performed in two dogs with PNP [109, 110]. Indirect immunofluorescence showed that both dogs had high titers of circulating anti-keratinocyte-IgG AA using canine lip [109] and canine gingiva, as well as anti-urothelial cell-IgG using urinary bladder [110] as substrates. Direct immunofluorescence revealed intercellular epidermal IgG in one dog (unpublished data) [110]; two follow-up studies using sera from this dog supported the involvement of anti-DSG3 IgG in the dissociation of keratinocytes in canine PNP [6, 88]. Both dogs [110, 120] also had IgG that targeted periplakin and envoplakin, as determined by immunoprecipitation or immunoblotting. Additional antigens recognized by AA from one dog included desmoplakin I and II, and the bullous pemphigoid antigen 1 [110]. Additional 130, 170 and 250 kDa bands, which were not characterized, were also found using immunoprecipitation in the second dog [110, 120]. All three dogs had a diagnosis of internal malignancy: a thymoma [27], a thymic lymphoma [109] and a splenic sarcoma [110].

In the only cat diagnosed with PNP, DIF was positive for IgG in the lower epidermis and IIF showed positive anti-keratinocyte and anti-urothelial cell IgG using normal feline buccal mucosal and canine bladder mucosa, respectively [111]. The identification of antigen(s) targeted by these AA was not performed. This cat was diagnosed with lymphocytic thymoma.

Altogether, these findings indicate that the pathogenesis of canine and, perhaps, feline PNP might be similar to the human disease homologue.

#### Signalment

In humans, PNP usually occurs between 45 and 70 years of age, with no sex predisposition to develop the disease [117]; it has also been reported in children and adolescents [121]. In another study of 104 patients with PNP, the affected age ranged from 11 to 83 years (average: 56.7), with a female-to-male ratio of 1.7 [122].

In animals, the breeds reported in dogs with PNP were a boxer, a bouvier des Flandres and a golden retriever [27, 109, 110]; these were two females and one male. When presented to the veterinarian, their ages were between 7 to 11-year-old. The only cat reported with PNP was an 8-year-old female spayed Himalayan.

#### Clinical signs

Human patients with PNP usually present with severe systemic signs (e.g., malaise, weakness and weight loss) in addition to skin and/or mucosal lesions [117]. The main characteristic feature of human PNP, and often the first presenting sign, is a stomatitis that presents as erosions and ulcers affecting the oropharynx and extending to the vermilion border of the lips [113, 117]. Indeed, mucosal and mucocutaneous regions (i.e., oral, conjunctiva, anogenital) are most commonly affected in patients with PNP [113, 117]. The skin lesions of PNP are polymorphic, and they can develop days, weeks of months after the onset of mucosal lesions [117]; these lesions may resemble those of EM (i.e., targetoid erythematous papules with central blisters), PV (i.e., flaccid blisters that rupture and form erosions and/or ulcers), or even graft-versus-host disease (i.e., erythematous-to-violaceous papules and plaques with silvery scale) [113, 117]. Lesions restricted to mucosae also have been reported in human PNP [113].

**Fig. 10:**
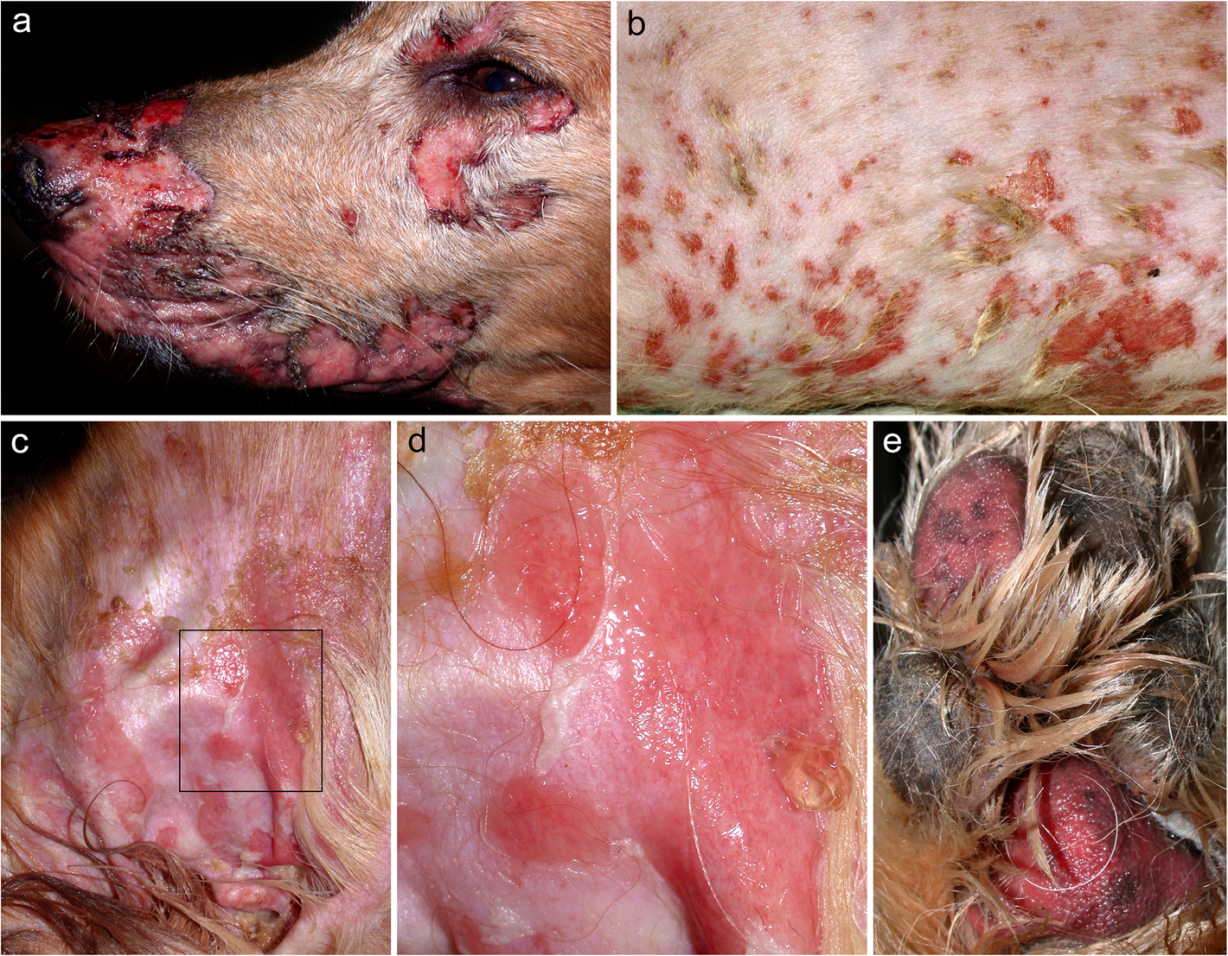
Clinical characteristics of canine paraneoplastic pemphigus. a-e: generalized erosive and ulcerative dermatitis affecting the face (**a**), trunk (**b**), pinnae (**c**-**d**) and footpads (**e**). The epidermis is seen sloughing from the pinna (**d**) and footpads (**e**) – **a**-to-**e** were taken from the same golden retriever (courtesy of Thierry Olivry; case reported in reference [106])

The three dogs with PNP had mucosal involvement [27, 109, 110], with two also exhibiting concurrent cutaneous lesions (Fig. 10) [109, 110]. In one dog [109], these developed after the onset of mucosal lesions; in another, both mucosal and cutaneous lesions had appeared concurrently [110]. The dog in Stannard’s report had only oral mucosal lesions [27].

The oral cavity and lips were the most common mucosae affected (3/3 dogs; 100%), followed by periocular mucocutaneous junctions (2/3 dogs; 67%) [109, 110]. The former feature is similar to that in human PNP. In the oral cavity, the tongue and gingiva were affected in all three dogs. The other affected mucosae included the prepuce and anus [110]. Nail bed involvement was reported in one dog [109]. The affected cutaneous regions were the nose, pinnae, muzzle, trunk (dorsal trunk, ventral thorax, axilla, groin) and footpads [109, 110].

The most common lesion of canine PNP are erosions/ulcers (3/3 dogs; 100%). Other lesions were vesicles (1/3 dogs; 33%) [109] and alopecia (1/3 dogs; 33%) [110]. Systemic signs were reported in all three dogs [27, 109, 110], and they were hyperthermia, lethargy, depression, anorexia, weight loss, and/or hypersalivation.

**Fig. 11:**
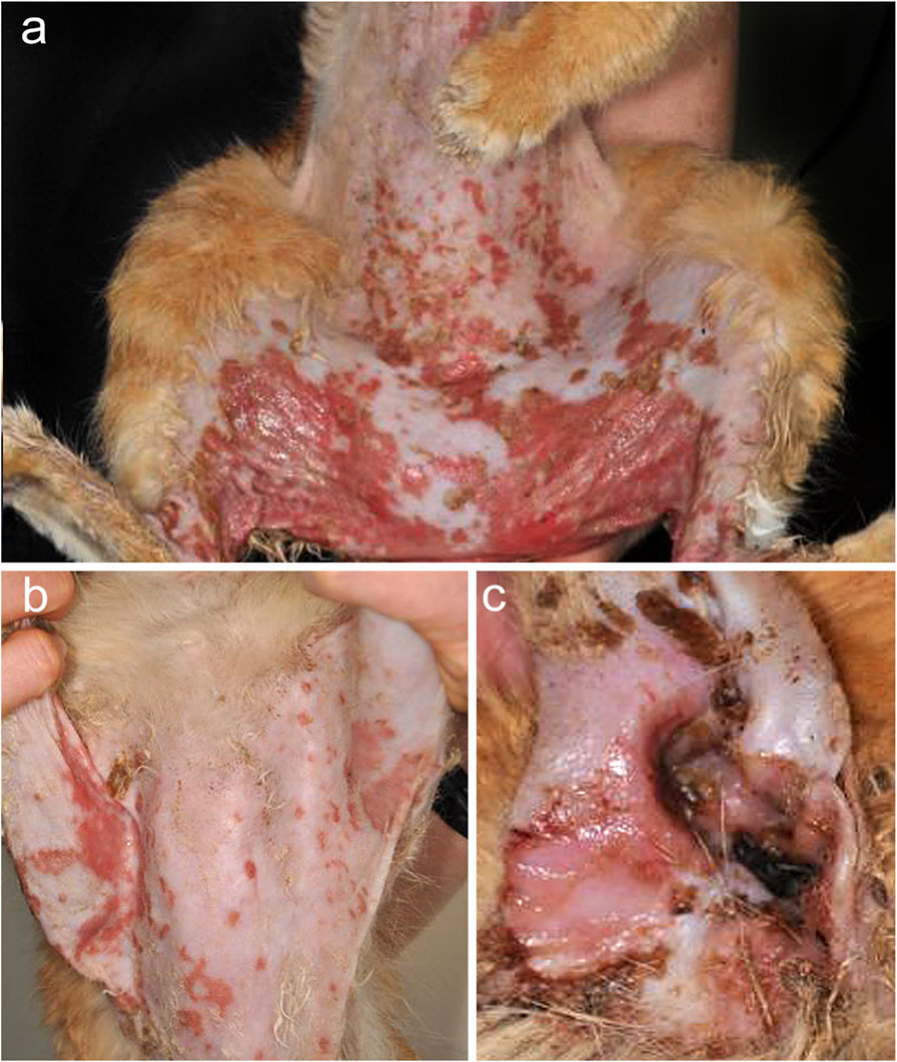
Clinical characteristics of feline paraneoplastic pemphigus. **a**-**c**: generalized erosive dermatitis affecting the abdomen (**a**), axillae and sternum (**b**), and pinna (**c**) in an Himalayan cat (Fig. 1 from reference [107], reprinted with permission from John Wiley and Sons under the license number 4754640544251)

The cat with PNP had skin lesions on the axillae, sternum, ventral abdomen, and perineum [111], and these were erosions, ulcers, crusts, and maculopapular eruptions (Fig. 11). This cat also had a thymoma-associated myasthenia gravis (MG) manifested by skeletal muscle weakness and a drooping mouth. There is only one case report of human PNP without mucosal involvement [123] and, interestingly, this patient also had a thymoma-associated MG.

#### Histopathology

**Fig. 12:**
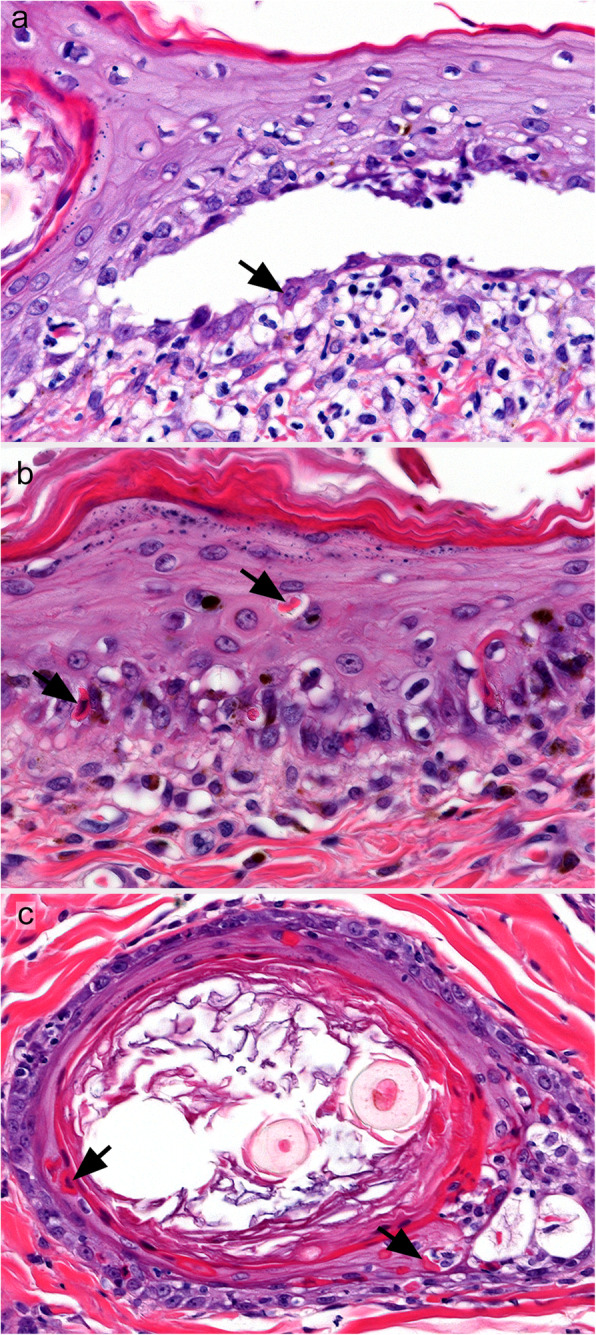
Histological characteristics of canine paraneoplastic pemphigus. **a**: an acantholytic suprabasal cleft in the epidermis retains a single row of basal keratinocytes (arrow) at its base, similar to pemphigus vulgaris. These basal cells are variably rounded or flattened - variation that is attributable to additional basal cell injury from concurrent interface dermatitis. Mild lymphocytic exocytosis occurs in the epidermis and the dermis has mixed neutrophilic and lymphocytic dermatitis; **b** and **c**: a lymphocytic cytotoxic dermatitis targets the basal layer (interface dermatitis) and suprabasal layers of the epidermis (**b**) and hair follicle infundibulum (**c**), which is similar to erythema multiforme. Apoptotic keratinocytes (arrows) are at different epithelial levels and are associated with lymphocytic exocytosis. **b** and **c** are from the same dog as (**a**) but are from different skin areas. **a** and **b** – 40x magnification. **c** – 20x magnification. Hematoxylin and eosin. All pictures are courtesy of Keith E. Linder

Histologically, PNP in humans exhibits reaction patterns of both PV and EM. The consistent lesions are PV-type suprabasal acantholysis, EM-associated keratinocyte apoptosis (sometimes called dyskeratosis in the earlier literature [3]) at multiple epidermal levels, lymphocytic/vacuolar interface dermatitis, and leukocytic exocytosis [3, 4, 108, 124]. In the few animals reported, these histological changes occurred in the skin and/or mucosa, as in humans (Fig. 12) [27, 109, 110]. Also like in humans, suprabasal acantholysis may, or may not, occur concurrently in the same biopsy as those of EM-type changes [109, 124]. When these two reaction patterns occur together, the associated tissue injury of one can disrupt the morphology of the other, potentially complicating the diagnosis. Interface dermatitis damages basal cells and leads to a tattered row of “tombstones” with loss and flattening of some acantholytic basal cells. Rupture of PV-type vesicles removes the upper epidermis and epidermal apoptosis along with it; although, apoptosis occurs at vesicle margins in some lesions. Prominent leukocytic exocytosis occurs in humans and includes lymphocytes, neutrophils, and eosinophils, while only lymphocytes and neutrophils have been described in dogs and in one cat, the severity of which varied. In animals, and in humans, lymphocytic satellitosis of apoptotic keratinocytes occurs and, in one dog, neutrophilic satellitosis was also described [109]. Lymphocytic interface dermatitis is considered cell-rich, based on the abundance of lymphocytic exocytosis and satellitosis; however, a band-like infiltrate below the epidermis is not always present. In the mucosa and perimucosal tissues, a band-like infiltrate can be expected as a generic inflammatory reaction of these tissue locations.

It should be noted that the apoptosis of keratinocytes appears to occur adjacent to suprabasal clefts in some cases of PV (personal observations of canine material) [31, 42] and thus PV can mimic PNP histologically. To help differentiate these disease, EM-type interface dermatitis and keratinocyte apoptosis in PNP should be sought in skin lesions without suprabasal clefts. Multiple biopsies could be needed to identify this feature. Results from immunologic studies, such as the demonstration of circulating anti-urothelium-IgG AA by IIF on bladder tissue and/or evidence of envoplakin and periplakin-targeting AA, can help to separate PNP from PV. The identification of neoplasia is required to establish a paraneoplastic syndrome; however, the clinical presentation, histopathology, and immunopathology are required to establish the diagnosis of PNP itself.

In humans and animals, a lymphocytic interface dermatitis and an acantholytic pustular dermatitis can also occur in other conditions. For example, we reported recently two dogs with poly-autoimmunity presented with comorbid PF and generalized cutaneous lupus erythematosus [125]. The co-occurrence of PF and discoid lupus erythematosus (DLE) occurs rarely in humans, and this is to be differentiated from pemphigus erythematosus (PE), which is a mild, facial form of PF with lesions clinically, but not histologically, resembling a DLE-associated malar rash [3, 126]. In veterinary medicine, PE is considered a pustular, erosive, and crusting disease of the face with clinical and histological features of PF and DLE [8]. Additionally, in animals with interface dermatitis of different causes, secondary superficial bacterial pyoderma might occur and produce acantholytic pustules. Fortunately, these conditions are easily be differentiated from PNP, because the clinical presentations and skin lesions are different and the histology lacks the PV-type suprabasal acantholysis.

#### Treatment and outcome

The prognosis of PNP in humans is poor and its mortality rate is high [113], the latter ranging from 75 to 90% of patients with the mean survival rate of less than 1 year [127]. Mortality usual results from severe infections due to immunosuppressive therapy, effects of the associated malignancy, or bronchiolitis obliterans. The latter is a condition caused by bronchial epithelial desquamation that results from (anti-plakin?) AA-induced acantholysis and sloughing of the epithelium into the airway lumen, which can lead to obstructive respiratory failure [113, 117]. Human PNP associated with benign tumors, such as localized Castleman’s disease or benign thymomas, has a better prognosis, as the CR of all lesions can be achieved within 1 to 2 years after tumor resection [113].

There is currently no standard therapy for human PNP [113, 117], and treatment is individualized based on the underlying malignancy, presence of comorbidities, and any adverse effects of immunosuppressants [117]. One of the key elements in treating human PNP is the complete excision of the solid tumor, and/or control of the associated hematological neoplasia [117] – the latter being the most common neoplasia associated with PNP in humans [113]. The most widely used therapy for human PNP is systemic GCs [113]. Cutaneous lesions tend to respond better to GC monotherapy [117], but mucosal lesions are more resistant and require a combination therapy with adjunct immunosuppressants such as AZA, MMF, or CYC [113, 117]. More recently, targeted therapy with the anti-CD20 monoclonal antibody rituximab, and the tyrosine kinase inhibitor ibrutinib have emerged as new treatment options with promising outcomes and few adverse effects [113].

In all three dogs, the PNP had a fatal outcome. Two dogs died naturally, with the cause of death presumed to be due to the underlying malignancy in one dog [27], and cardiac abnormalities 12 h post-surgery (performed to remove the splenic sarcoma) in the other [110]; medical therapy for PNP had not been initiated in these two dogs that died [27, 110]. The last dog was euthanized due to a lack of response to treatment, which consisted of high-dose oral prednisolone [109].

In the single case report of feline PNP, a CR was achieved with the complete surgical removal of the lymphocytic thymoma, along with high-dose prednisolone and chlorambucil [111]. The CR was obtained in 4 months, and the cat’s PNP continued to be in remission for the following 4 years without any therapy. Interestingly, the anti-keratinocyte IgG AA titre, measured at 7 months into CR, was 1:10 as compared to > 1:160 at the time of diagnosis, suggesting that the disease severity and autoantibody titres could be correlated.

#### Implications for practice

The immediate therapeutic goal for PNP, whenever possible, should be the complete removal or remission induction of the underlying malignancy. If not possible or achievable, intervention(s) should be aimed at controlling the underlying neoplasia, along with the management of the mucosal and/or skin lesions associated with PNP. Therefore, a multidisciplinary approach is crucial and general practitioners should work together with a veterinary oncologist and dermatologist to manage the patient.

With this in mind, the rapid diagnosis of either the underlying neoplasia, or PNP is vital for a more favorable outcome. Because diagnostic lesions are fragile and because biopsies need to capture both EM-type and PV changes, multiple skin biopsies should be collected in addition to biopsies of any oral cavity lesions. When presented with a dog or cat in which PNP has been confirmed, an extensive and a thorough diagnostic work-up should be started to detect any underlying neoplasia. There is too little information on the most effective therapy for canine and feline PNP, but by extrapolating data from the human disease, it is reasonable to start a combination therapy of oral GC and adjunct immunosuppressants (i.e., AZA for dogs and chlorambucil for cats). However, pet owners should be informed that the prognosis for canine and feline PNP is poor, especially if the underlying neoplasia is malignant and cannot be treated.

#### Implications for research

Due to the grave prognosis of PNP, the early detection of either PNP or the underlying neoplasia to allow for immediate removal of the tumor could potentially be life-saving. The development of an ELISA screening test for the detection of envoplakin and/or periplakin in canine and/or feline species could shorten the time for diagnosis; these two plakin family proteins were detected in the two dogs with PNP and could be more specific than DSG1 or 3.

It is a mystery as to why so many animals with thymoma do not develop PNP. For instance, in one recent study of 116 dogs with this tumor, only one had cutaneous lesions diagnosed as EM based on histopathology [128]. Could this reason be that some dogs have a genetic predisposition that “allows” the thymoma to produce AA (i.e., breakdown of central and/or peripheral immune tolerance [113]? Or, could it be that the thymoma is detected and removed early before AA are produced? These unanswered questions warrant further investigation.

A collaboration of general practitioners, veterinary dermatologists, oncologists, and immunologists is essential to gather more information and understand PNP better. This could begin with a multicenter collaboration for the identification of cats and dogs with PNP with the pooled data used for a better characterization of this disease.

## Conclusion

There are striking similarities of the deep pemphigus disease (PV, PVeg and PNP) between humans and animals. These chronic and often-relapsing autoimmune dermatoses remain a therapeutic challenge. Over the last two decades, there has been tremendous progress with the development of newer targeted therapies (i.e., rituximab, alemtuzumab) [117] that have resulted in a safer and more effective treatment for humans with these devastating diseases. Veterinary clinicians and scientists could learn from their human counterparts and continue their efforts to improve the diagnosis and management of deep pemphigus variants in animals.

## Data Availability

This article being a review of published information, data sharing is not applicable as no datasets were generated or analyzed.

## Abbreviations

AA: Autoantibodies
AZA: Azathioprine
CR: Complete remission
CsA: Ciclosporin, cyclosporine, cyclosporin
CPS: Cutaneous paraneoplastic syndrome
CYC: Cyclophosphamide
DSC: Desmocollin
DSG: Desmoglein
DIF: Direct immunofluorescence
DSH: Domestic shorthaired
ELISA: Enzyme-linked immunosorbent assay
EM: Erythema multiforme
FDA: Food and Drug Administration
GC: Glucocorticoid
HLA: Human leukocyte antigen
IgG: Immunoglobulin G
IIF: Indirect immunofluorescence
IV: Intravenous
MAB: Monoclonal antibody
MG: Myasthenia gravis
MMF: Mycophenolate mofetil
NYSCVM: New York State College of Veterinary Medicine
PF: Pemphigus foliaceus
PR: Partial response
PV: Pemphigus vulgaris
PVeg: Pemphigus vegetans
PNP: Paraneoplastic pemphigus
RTX: Rituximab
SLO: Symmetric lupoid onychodystrophy
Th: T helper
TNF: Tumor necrosis factor
